# Molecular characteristics of carbapenem-resistant *Acinetobacter* spp*.* from clinical infection samples and fecal survey samples in Southern China

**DOI:** 10.1186/s12879-019-4423-3

**Published:** 2019-10-28

**Authors:** Si Li, Xiaonv Duan, Yuan Peng, Yongyu Rui

**Affiliations:** Laboratory Medicine Center, Nanfang Hospital, Southern Medical University, Tonghe, Guangzhou, 510515 China

**Keywords:** *Acinetobacter*, Carbapenem resistance, Colistin, Fecal

## Abstract

**Background:**

Carbapenem resistance among *Acinetobacter* species has become a life-threatening problem. As a last resort in the treatment of gram-negative bacteria infection, resistance to colistin is also a serious problem. The aim of study was to analyze the mechanism of resistance and perform genotyping of carbapenem-resistant *Acinetobacter* from clinical infection and fecal survey samples in Southern China.

**Methods:**

One hundred seventy and 74 carbapenem-resistant *Acinetobacter* were isolated from clinical infection samples and fecal survey samples, respectively. We detected the related genes, including carbapenemase genes (*bla*_KPC_, *bla*_IMP_, *bla*_SPM_, *bla*_VIM_, *bla*_NDM_, *bla*_OXA-23-like_, *bla*_OXA-24/40-like_, *bla*_OXA-51-like_, and *bla*_OXA-58-like_), colistin resistance-related genes (*mcr-1*, *mcr-2*, *mcr-3*, *mcr-4*, and *mcr-5*), a porin gene (*carO*), efflux pump genes (*adeA*, *adeB*, *adeC*, *adeI*, *adeJ*, and *adeK*), mobile genetic element genes (*intI1*, *intI2*, *intI3*, *tnpU*, *tnp513*, IS*26*, IS*Aba1*, and IS*Aba125*), and the integron variable region. Genotyping was analyzed by enterobacterial repetitive intergenic consensus (ERIC)-PCR and dendrogram cluster analysis.

**Results:**

Among the 244 carbapenem-resistant *Acinetobacter*, the common carbapenemase-positive genes included the following: *bla*_OXA-51-like_, 183 (75.00%); *bla*_OXA-23-like_, 174 (71.30%); *bla*_NDM-1_, 57 (23.40%); and *bla*_OXA-58-like_, 30 (12.30%). The coexistence of *mcr-1* and *bla*_NDM-1_ in five strains of *A*. *junii* was found for the first time. Eleven distinct *carO* gene variants were detected in 164 (67.20%) strains, and ten novel variants, which shared 92–99% identity with sequences in the Genbank database, were first reported. Efflux system genes were present in approximately 70% of the isolates; *adeABC* and *adeIJK* were observed in 76.23 and 72.13%, respectively. Class 1 integrons were detected in 180 (73.80%) strains and revealed that four gene cassette arrays contained 11 distinct genes. The genotyping by ERIC-PCR demonstrated a high genetic diversity of *non-baumannii Acinetobacter*, and greater than 90% similarity to *A*. *baumannii*.

**Conclusions:**

The *bla*_NDM-1_ gene was identified in up to 77% of the carbapenem-resistant *Acinetobacter* isolated from fecal survey samples, indicating that the gut might be a reservoir of resistant opportunistic bacteria. Intestinal bacteria can be transmitted through the fecal-hand, which is a clinical threat, thus, the monitoring of carbapenem-resistant bacteria from inpatients’ feces should be improved, especially for patients who have been using antibiotics for a long time.

**Electronic supplementary material:**

The online version of this article (10.1186/s12879-019-4423-3) contains supplementary material, which is available to authorized users.

## Background

*Acinetobacter* species are a heterogeneous group of strictly aerobic, non-motile gram-negative, non-fermenting encapsulated coccobacilli that can be found in the environment [1, 2]. Among them, *Acinetobacter baumannii* has become a major cause of nosocomial infections in recent years. *A*. *baumannii* is a conditional pathogen, especially when the resistance of inpatients declines; *A*. *baumannii* can invade the human body in various ways and cause fatal infection [3, 4]. Meanwhile, infection caused by *Acinetobacter* species other than *A*. *baumannii* has caused concern recently [5]. Moreover, the developed resistance of *Acinetobacter* to a variety of commonly used antimicrobials has led to ineffective antibiotics and increased mortality following infection [6]. Carbapenems are commonly used to treat infections caused by *Acinetobacter* species; an outbreak of carbapenem-resistant *Acinetobacter* species can complicate therapy and may lead to treatment failure [7, 8].

There are three main mechanisms that *Acinetobacter* species use to resist carbapenems: production of enzymes (e.g., carbapenemase) that are capable of hydrolyzing carbapenems; altered function of membrane-associated proteins such as porins; and activation of drug efflux pumps [9]. Carbapenemases that have been reported in *Acinetobacter* species include Ambler class B metallo-β-lactamases, such as IMP and VIM, and the recently described NDM; Ambler class D oxacillinases (OXAs); and Ambler class A β-lactamases [10, 11]. The OXA carbapenemase genes of *Acinetobacter* species are divided into some phylogenetic subgroups: *bla*_OXA-23-like_, *bla*_OXA-24/40-like_, *bla*_OXA-51-like_, *bla*_OXA-58-like_, *bla*_OXA-143-like_, and *bla*_OXA-235-like_. Although OXA carbapenemase weakly hydrolyze carbapenems, they can confer higher resistance when *bla*_OXA_ genes are overexpressed using a strong promoter with mobile insertion elements such as IS*Aba1* [12, 13]. Low outer membrane permeability, due to changes in the primary structure or loss of carbapenem-associated outer membrane protein (*carO*), a 25/29-kDa outer-membrane protein, is one of the best-characterized mechanisms of intrinsic carbapenem resistance in *Acinetobacter* species [14, 15]. Drug efflux pumps are most often associated with carbapenem resistance in *Acinetobacter* species when overexpressed; the resistance-nodulation-division (RND)-type family is of particular clinical importance [16]. To date, five RND efflux pumps have been described in *Acinetobacter* species: AdeABC, AdeDE, AdeIJK, AdeXYZ, and AdeFGH [17].

Colistin has been used in veterinary and human medicine for over 50 years. Colistin has broad-spectrum activities against gram-negative bacteria, but is associated with both nephrotoxicity and neurotoxicity, limiting its clinical application. However, colistin has now emerged as an effective therapeutic against carbapenem-resistant bacteria. Now, over-reliance on colistin to treat these diseases has led to the development of resistance to colistin [18]; thus, exploring the resistance mechanism of *Acinetobacter* species will be important.

*Mcr*, a plasmid-mediated colistin resistance gene first reported in 2015 in China [19], has increased our knowledge of colistin-resistance mechanisms other than chromosomal mechanisms. The plasmid-borne colistin-resistance gene *mcr* can also be transmitted and transferred by mobile genetic components [20].

Mobile genetic elements, such as integrons, transposons, and insertion sequences, provide bacteria with a powerful genetic toolbox, which could lead to multidrug-resistant (MDR) strains. Transfer of mobile genetic elements between different isolates, even in different species, has also been described in *Acinetobacter* [21, 22].

Various bacteria, including MDR bacteria, are contained in the gut microbiota, which serves as an important and large reservoir of resistance genes. Fecal samples might therefore be the ideal specimen for studying and detecting antimicrobial-resistant genes. Several studies have reported high rates of fecal carriage for *Acinetobacter*, making the digestive tract a potential reservoir for nosocomial infections and outbreaks [23]. The aim of this study was to analyze the mechanism of resistance and perform genotyping of carbapenem-resistant *Acinetobacter* from clinical infection and fecal survey samples in Southern China.

## Methods

### Bacterial isolates and species identification

Between January 2014 and December 2015, 170 non-duplicate carbapenem-resistant *Acinetobacter* isolates were obtained from clinical infection samples (including sputum, puncture fluid, urine, instrument, blood) which were collected from Nanfang Hospital, a large general and teaching hospital in Guangzhou, China.

Moreover, 5000 fecal survey samples were collected from July 2014 to June 2015, followed by incubation on MacConkey agar plates with 2 μg/mL meropenem. A total of 434 g-negative carbapenem-resistant isolates were collected. The *recA* [24] gene was amplified from non-fermenting isolates and used as a control for the genus *Acinetobacter*. Finally, 74 carbapenem-resistant *Acinetobacter* species were obtained. The study was approved by the Medical Ethics Committee of Nanfang Hospital Southern Medical University and conducted in compliance with the Declaration of Helsinki.

All isolates were confirmed as *Acinetobacter* species by biochemical methods, the *recA* gene, and analysis of 16S ribosomal RNA (rRNA) sequences. The *Acinetobacter calcoaceticus*–*A*. *baumannii* complex was confirmed by 16–23S rRNA and internal transcribed spacer sequences [25].

### Antimicrobial susceptibility testing

Analysis of susceptibility to antimicrobial compounds was performed using the automated BD Phoenix 100 Microbiology System (Becton Dickinson and Co., Franklin Lakes, NJ, USA) according to the guidelines of the Clinical and Laboratory Standards Institute (CLSI) M100-S27 criteria. The antibiotics tested were ampicillin, ampicillin-sulbactam, amoxicillin-clavulanic acid, piperacillin, piperacillin-tazobactam (4 mg/L tazobactam), ceftazidime, cefotaxime, cefazolin, cefepime, imipenem, meropenem, aztreonam, chloramphenicol, levofloxacin, ciprofloxacin, gentamicin, tetracycline, colistin, trimethoprim-sulfamethoxazole, and amikacin. *Escherichia coli* ATCC 25922 and *Pseudomonas aeruginosa* ATCC 27853 were used as control strains for antimicrobial susceptibility testing.

### Molecular detection of resistance genes

DNA was extracted using the AccuPrep® genomic DNA extraction kit according to the manufacturer’s instructions. PCR was performed for antibiotic resistance-related genes described below.

The following carbapenemase-encoding genes were detected: class A β-lactamases genes: *bla*_KPC_ [26]; class B metallo-β-lactamases genes: *bla*_IMP_ [26], *bla*_VIM_ [26], *bla*_SPM_ [26], and *bla*_NDM_ [26]; and class D oxacillinases genes: *bla*_OXA-23-like_ [27], *bla*_OXA-24/40-like_ [27], *bla*_OXA-51-like_ [27], and *bla*_OXA-58-like_ [27]. The colistin-related resistance gene: *mcr-1* [28], *mcr-2* [28], *mcr-3* [28], *mcr-4* [28], and *mcr-5* [28]*.* The protein-related gene *carO* [29] (*A*. *baumannii* outer membrane protein involved in carbapenem resistance) and the drug efflux pump component-related genes *adeA* [30], *adeB* [31], *adeC* [31], *adeI* [31], *adeJ* [31], and *adeK* [31] were also detected.

Database searches for DNA sequence similarities were performed using BLAST (https://blast.ncbi.nlm.nih.gov). The construction of a *carO* phylogenetic tree was obtained by MEGA using the neighbor-joining method. A model of the p-distance was used to estimate distances for nucleotide sequences. The significance of the groups observed in constructed trees was determined by bootstrap analysis with 1000 replicates.

Mobile genetic element-related resistance genes, including integrons (*intI1* [32, 33], *intI2* [32, 34], *intI3* [32]), transposons (*tnpU* [35] and *tnp513* [35]), the variable region of class 1 integron and class 2 integron, and insertion sequences (*IS26* [36], *ISAba1* [37], and *ISAba125* [37]) were also analyzed. The PCR cycle consisted of denaturation at 94 °C for 5 min, followed by 35 cycles of 30 s at 94 °C, annealing for 40 s at optimal annealing temperature, and extension at 72 °C for 40 s for the genes and 4 min for the variable region of intI1 and intI2.

The primers used for PCR are shown in Table 1. Primer synthesis and sequencing of PCR products were performed by Sangon Biotech Co., Ltd. (Shanghai, China).

**Table 1:** Gene primers used for PCR amplification of genes associated with antimicrobial resistance

Gene.	Primer sequence(5′-3′)		Product (bp)	T_m_(°C)	Reference
	Forward	Resever			
Identification					
*recA*	CCTGAATCTTCTGGTAAAAC	GTTTCTGGGCTGCCAAACATTAC	405	56	[23]
16S rRNA	CCAGCAGCCGCGGTAATACG	ATCGGYTACCTTGTTACGACTTC	approximately 996	55	
16-23S rRNA ITS	GTCGTAACAAGGTAGCCGTA	GGGTTYCCCCRTTCRGAAAT	variable	60	[24]
ERIC-PCR	AAGTAAGTGACTGGGGTGAGCG		/	58	
class A β-lactamases					
*bla*_KPC_	CGT CTA GTT CTG CTG TCT TG	CTT GTC ATC CTT GTT AGG CG	798	60	[25]
Class B metallob-β-lactamases					
*bla*_IMP_	GGA ATA GAG TGG CTT AAY TCT C	GGT TTA AYA AAA CAA CCA CC	232	50	[25]
*bla*_VIM_	GAT GGT GTT TGG TCG CAT A	CGA ATG CGC AGC ACC AG	390	58	[25]
*bla*_SPM_	AAA ATC TGG GTA CGC AAA CG	ACA TTA TCC GCT GGA ACA GG	271	55	[25]
*bla*_NDM_	GGT TTG GCG ATC TGG TTT TC	CGG AAT GGC TCA TCA CGA TC	621	60	[25]
class D oxacillinases					
*bla*_OXA-23-like_	GAT CGG ATT GGA GAA CCA GA	ATT TCT GAC CGC ATT TCC AT	501	53	[26]
*bla*_OXA-24-like_	GGT TAG TTG GCC CCC TTA AA	AGT TGA GCG AAA AGG GGA TT	246	53	[26]
*bla*_OXA-51-like_	TAA TGC TTT GAT CGG CCT TG	TGG ATT GCA CTT CAT CTT GG	353	54	[26]
*bla*_OXA-58-like_	AAG TAT TGG GGC TTG TGC TG	CCC CTC TGC GCT CTA CAT AC	599	54	[26]
Colistin-related resistance gene					
*mcr-1*	AGTCCGTTTGTTCTTGTGGC	AGATCCTTGGTCTCGGCTTG	320	58	[27]
*mcr-2*	CAAGTGTGTTGGTCGCAGTT	TCTAGCCCGACAAGCATACC	715	58	[27]
*mcr-3*	AAATAAAAATTGTTCCGCTTATG	AATGGAGATCCCCGTTTTT	929	58	[27]
*mcr-4*	TCACTTTCATCACTGCGTTG	TTGGTCCATGACTACCAATG	1116	58	[27]
*mcr-5*	ATGCGGTTGTCTGCATTTATC	TCATTGTGGTTGTCCTTTTCTG	1644	58	[27]
porins					
*carO*	ATT GTA GAA AGC TGA GAC AT	ATT TCT YTA TGC TCA CCT GA	variable^a^	50	[28]
drug efflux pumps					
*adeA*	GCTGAGCCACCACCGGCTAAAG	ACCTTCAACAACGACTCTGTCACC	990	56	[29]
*adeB*	CTTGCATTTACGTGTGGTGT	GCTTTTCTACTGCACCCAAA	168	54	[30]
*adeC*	CCCAACCATTGGTGTAACG	GAACATCCGTGCTTTAGC	626	52	[30]
*adeI*	CAAATGCAAATGTAGATCTTGG	AAACTGCCTTTACTTAGTTG	210	51	[30]
*adeJ*	GGTCATTAATATCTTTGGC	GGTACGAATACCGCTGTCA	221	55	[30]
*adeK*	TTGATAGTTACTTGACTGTTC	GGTTGGTGAACCACTGTATC	162	51	[30]
integrase					
*intI1*	GCA TCC TCG GTT TTC TGG	GGT GTG GCG GGC TTC GTG	457	60	[32]
variable region	GGCATCCAAGCAGCAAG	AAGCAGACTTGACCTGA	variable	52	[33]
*intI2*	CAC GGA TAT GCG ACA AAA AGG T	GTA GCA AAC GAG TGA CGA AAT G	789	54	[32]
variable region	GTAGCAAACGAGTGACGAAATG	GAATTCGACATGTTTGGACGCCTTGGC	variable	52	[33]
*intI3*	ATC TGC CAA ACC TGA CTG	CGA ATG CCC CAA CAA CTC	922	60	[32]
transposon					
*tnpU*	CCAACTGATGGCGGTGCCTT	CGGTATGGTGGCTTTCGC	403	59	[35]
*tnp513*	ATGTCGCTGGCAAGGAACGC	GGGTTCGCTGCGAGGATTGT	240	59	[35]
Insertion sequence					
IS*26*	AGCGGTAAATCGTGGAGTGA	CAAAGTTAGCGATGAGGCAG	619	58	[36]
IS*Aba1*	CACGAATGCAGAAGTTG	CGACGAATACTATGACAC	550	55	[37]
IS*Aba125*	GGGTAATGCTCGTATCGT	TAGACGTAGACGTGGTCA	184	51	[37]

^a^The forward primer hybridizes to a region located 294 bp upstream of the 5′ end of the *carO* initiation codon, while the reverse primer anneals to a sequence located 131 bp downstream of the *carO* termination codon

### Eric-PCR

Enterobacterial Repetitive Intergenic Consensus (ERIC-PCR) was performed on all isolates depending on the different species [38]. Band comparisons were performed by clustering analysis based on the unweighted pair-group method with arithmetic mean of isolates using Quantity One-v4.6.7.

## Results

### Identification of isolates

During the study period, 244 (170 from clinical infection samples and 74 from fecal survey samples) carbapenem-resistant *Acinetobacter* species were collected. PCR amplification of the *recA* gene of all strains was positive. Further identification revealed 181 *A*. *baumannii* and 63 non-*baumannii Acinetobacter* species. Remarkably, clinical infection samples and fecal survey samples exhibited large differences in species (Table 2). *Acinetobacter baumannii* was the predominant clinical carbapenem-resistant bacteria (97.1%). However, 58 carbapenem-resistant *non-baumannii Acinetobacter* were isolated in fecal survey samples, and 77.59% were further identified as *A*. *junni*.

**Table 2:** Numbers of isolated *Acinetobacter* strains

Species	Clinical samples	Non-clinical fecal samples	Total
*A. baumannii*	165	16	181
*A. calcoaceticus*	2	2	4
*A. johnsonii*	0	7	7
*A. junii*	2	45	47
*A.gandensis*	0	1	1
*A.bereziniae*	0	3	3
*A. haemolyticus*	1	0	1
Total	170	74	244

### Antimicrobial susceptibility testing

According to the minimal inhibitory concentration (MIC) values of tested antibiotics (Table 3 and Additional file 1: Table S1), the MIC breakpoints of *A. baumannii complex* to colistin refer to the CLSI standard, and the MIC breakpoints of *non-baumannii Acinetobacter* to colistin refer to the EUCAST standard, because the CLSI standard was applicable to *A. baumannii complex* only. All isolates were resistant to carbapenems and cephems, including cefazolin, ceftazidime, and cefepime, and sensitivity to amikacin and colistin was observed in 32.80 and 97.10% of the isolates, respectively. However, *non-baumannii Acinetobacter* was more sensitive to amikacin than *Acinetobacter baumannii.* Six *non-baumannii Acinetobacter* and one *Acinetobacter baumannii* were resistant to colistin. Notably, all of the carbapenem-resistant *Acinetobacter* isolates were MDR (Table 3) [39].

**Table 3:** Susceptibilities of *Acinetobacter* species and the distribution of resistant genes among these species

	No.(%) of isolates:				
	clinical infection samples		clinical fecal survey samples		Total
	*A. baumannii* (*n* = 165)	non*-baumannii Acinetobacter* (*n* = 5)	*A. baumannii* (n = 16)	non*-baumannii Acinetobacter* (*n* = 58)	*Acinetobacter* species (*n* = 244)
Antimicrobial agent					
Amikacin**	20 (12.1)	1 (20.00)	9 (56.3)	50 (86.2)	80 (32.8)
Gentamycin**	1 (0.6)	0 (0)	5 (31.3)	13 (22.4)	19 (7.8)
Imipenem	0 (0)	0 (0)	0 (0)	0 (0)	0 (0)
Meropenem	0 (0)	0 (0)	0 (0)	0 (0)	0 (0)
Colistin*	164 (99.4)	5 (100)	16 (100)	53 (91.4)	238 (97.5)
Ciprofloksacin	17 (10.3)	0 (0)	6 (37.5)	6 (10.3)	29 (11.9)
Levofloxacin**	17 (10.3)	0 (0)	6 (37.5)	23 (39.7)	46 (18.9)
Tetracycline**	1 (0.6)	0 (0)	6 (37.5)	25 (43.1)	32 (13.1)
Trimethoprim-Sulfamethoxazole**	16 (9.7)	0 (0)	5 (31.3)	18 (31.0)	39 (16.0)
Carbapenemase-encoding genes					
class A β-lactamases					
*bla*_KPC_	0 (0)	0 (0)	0 (0)	0 (0)	0 (0)
class B metallob-β-lactamases					
*bla*_IMP_	1 (0.6)	0 (0)	0 (0)	1 (1.7)	2 (0.8)
*bla*_VIM_**	0 (0)	0 (0)	2 (12.5)	2 (3.4)	5 (2.0)
*bla*_SPM_	0 (0)	0 (0)	0 (0)	0 (0)	0 (0)
*bla*_NDM_**	0 (0)	0 (0)	2 (12.5)	55 (94.8)	57 (23.4)
class D oxacillinases					
*bla*_OXA-23-like_**	159 (96.4)	5 (100.0)	8 (50.0)	2 (3.4)	174 (71.3)
*bla*_OXA-24-like_**	0 (0)	0 (0)	5 (31.3)	1 (1.7)	6 (2.5)
*bla*_OXA-51-like_**	165 (100)	4 (80.0)	13 (81.3)	0 (0)	182 (74.6)
*bla*_OXA-58-like_**	6 (3.6)	0 (0)	0 (0)	24 (41.4)	30 (12.3)
colistin-related resistance gene					
*mcr-1*	0 (0)	0 (0)	0 (0)	5 (8.6)	5 (2.0)
*mcr-2*	0 (0)	0 (0)	0 (0)	0 (0)	0 (0)
*mcr-3*	0 (0)	0 (0)	0 (0)	0 (0)	0 (0)
*mcr-4*	0 (0)	0 (0)	0 (0)	0 (0)	0 (0)
*mcr-5*	0 (0)	0 (0)	0 (0)	0 (0)	0 (0)
porins					
carO**	144 (87.3)	3 (60.0)	15 (93.8)	2 (3.4)	164 (67.2)
drug efflux pumps					
adeA**	160 (97.0)	4 (80.0)	13 (81.3)	15 (25.9)	192 (78.7)
adeB**	160 (97.0)	4 (80.0)	15 (93.8)	18 (31.0)	197 (80.7)
adeC**	145 (87.9)	3 (60.0)	15 (93.8)	12 (20.7)	175 (71.7)
adeI**	143 (86.7)	3 (60.0)	12 (75.0)	6 (10.3)	164 (67.2)
adeJ**	100 (60.6)	3 (60.0)	9 (56.3)	8 (13.8)	120 (49.2)
adeK**	89 (54.0)	2 (40.0)	7 (43.8)	7 (12.1)	105 (43.0)
integron					
*intI1*	122 (73.9)	2 (40.0)	12 (75.0)	44 (75.9)	180 (73.8)
intI2**	0 (0)	0 (0)	0 (0)	5 (8.6)	5 (2.0)
*intI3*	0 (0)	0 (0)	0 (0)	0 (0)	0 (0)
transposon					
*tnpU*	112 (67.9)	1 (20.0)	8 (50.0)	33 (56.9)	154 (63.1)
tnp513*	119 (72.1)	4 (80.0)	9 (56.3)	34 (58.6)	166 (68.0)
insertion sequence					
IS*26*	159 (96.4)	5 (100.0)	16 (100.0)	58 (100.0)	238 (97.5)
IS*Aba1*	150 (90.9)	5 (100.0)	14 (87.5)	55 (94.8)	224 (91.8)
IS*Aba125*	154 (93.3)	5 (100.0)	14 (87.5)	57 (98.3)	230 (94.3)

*P < 0.05;**P < 0.01

* shows a difference between the results of clinical infection samples and clinical fecal survey samples

### Molecular detection of resistance genes

#### Carbapenemase-encoding genes

The prevalence of *bla*_OXA-23-like_, *bla*_OXA-24/40-like_, *bla*_OXA-51-like_, and *bla*_OXA-58-like_ gene occurrences were 71.31, 2.45, 75.00, and 12.30%, respectively. Co-existence of the *bla*_OXA-23-like_ and *bla*_OXA-51-like_ genes was detected in 171 strains. It is notable that three strains simultaneously carried the genes *bla*_OXA-23-like_, *bla*_OXA-51-like_, and *bla*_OXA-58-like_. With regard to the 181 isolates of *A*. *baumannii*, 179 (98.90%) strains carried the *bla*_OXA-51-like_ gene, which is characteristic of *A*. *baumannii*. For other carbapenemase genes in all strains, 2 (0.82%) were positive for the *bla*_IMP_ gene, 4 (1.64%) for the *bla*_VIM_ gene, and 57 (23.36%) for the *bla*_NDM_ gene. It is remarkable that all *bla*_NDM_-positive strains were isolated from fecal survey samples, while 41 strains possessed *bla*_NDM-1,_ 12 strains possessed *bla*_NDM-5_, and 4 strains possessed *bla*_NDM-4_. The genes *bla*_KPC_ and *bla*_SPM_ were not identified.

#### Colistin-related resistant genes

Six (8.70%) *non-baumannii Acinetobacter* and one (0.55%) *A*. *baumannii* isolate was resistant to colistin, while five *non-baumannii Acinetobacter* strains from fecal samples tested positive for the *mcr-1* gene.

#### Protein-related genes

The *carO* gene was detected in 164 (67.21%) strains, and an analysis of 164 complete *carO* porin nucleotide sequences was performed. All strains showed amplified products of approximately 1200 bp, with the exception of *A*. *baumannii* 100588A and 513,007, in which a band of approximately 2000 bp was amplified and the distal part of the insertion (from 1066 to 2259 of the sequence) showed 100% identity with a fragment from *A*. *baumannii* BJAB0715 (GenBank accession number CP003847.1, position 2960028 to 2961220). *Acinetobacter* sp. 1029087 (GenBank accession number KX517470.1) contained an insertion of 2 bp (starting at +230 of the *carO* gene), which generated a stop codon (TAA) at position 247–249 in the nucleotide sequence of the *carO* gene.

**Fig. 1:**
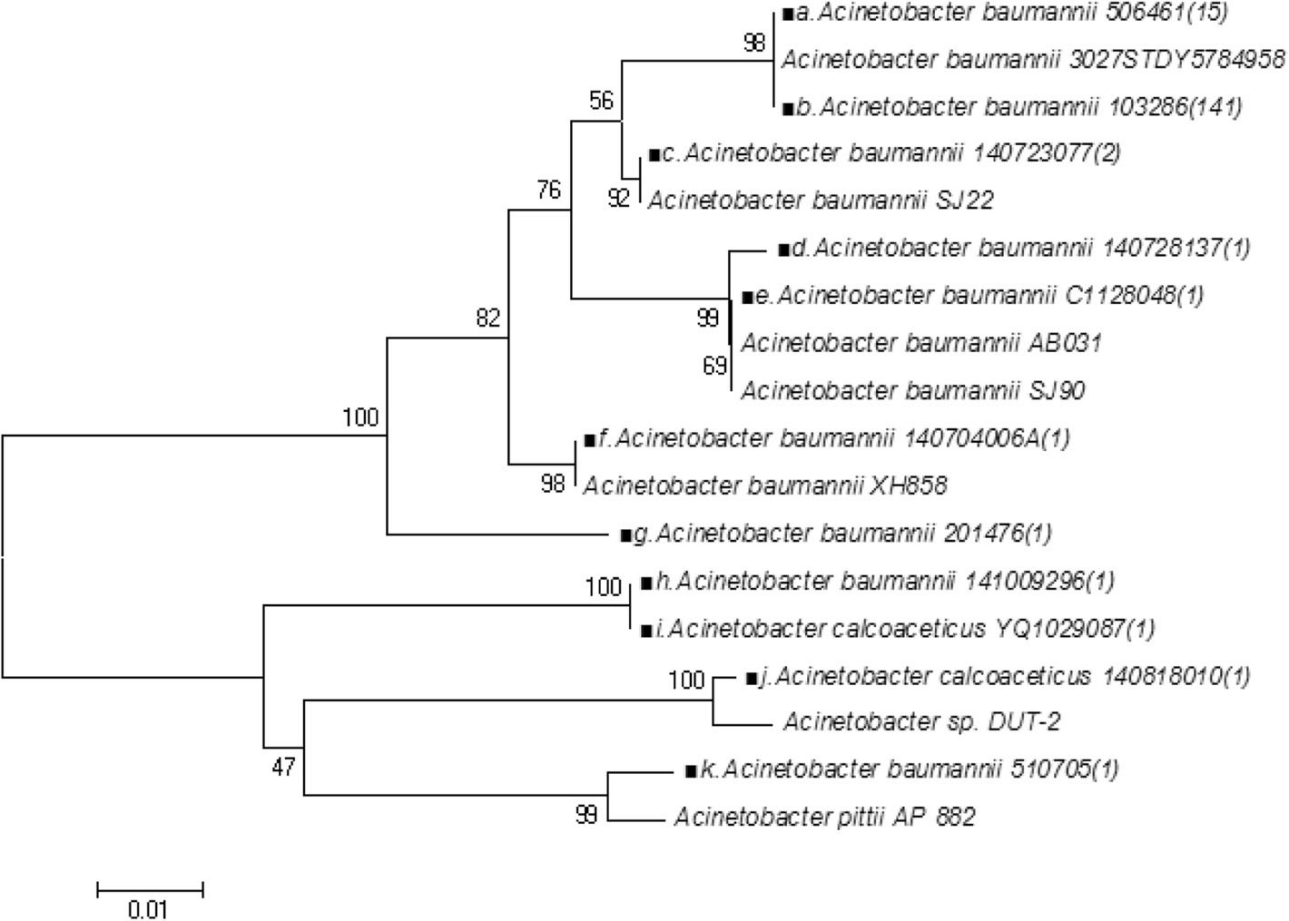
Phylogenetic inferences of the *carO* gene among *Acinetobacter* species. A phylogenetic tree of the *carO* gene was constructed by MEGA software using the neighbor-joining method. The numbers in parentheses correspond to the number of isolates within the genotype. The black block represents the variants detected in this study. The nucleotide sequences of the structure A to K in this work have been submitted to the GenBank database and assigned the following accession numbers: KX517486, LT594095, KX517462, KX517464, KX517472, KX517465, KX517458, KX517469, KX517470, KX517467, and KX517489

Sequencing of *carO* genes revealed the presence of 11 distinct variants; 10 novel sequences were discovered, which were designated as different if they had at least one nucleotide change. The dominant sequence was 99–100% identical to *A*. *baumannii* strain 3027STDY5784958 (GenBank accession number LT594095.1) and the ten novel sequences exhibited 92–99% identity with sequences in the database. The novel sequences were a result of deletions, insertions, or point mutations. Phylogenetic analysis of the detected *carO* gene showed that all sequences were classified into two groups (Fig. 1).

#### Drug efflux pump component-related genes

Efflux system genes were present in approximately 70% of isolates, and *adeABC* and *adeIJK* were observed in 76.23 and 72.13%, respectively.

#### Mobile genetic element-related resistance genes

Multidrug resistance to aminoglycosides, quinolones, tetracycline, and trimethoprim-sulfamethoxazole antimicrobial agents might be a result of the presence of a high rate of mobile genetic elements, including IS*26* (97.54%), IS*Aba1* (90.16%), IS*Aba125* (93.44%), *tnpU* (63.52%), and *tnp513* (68.03%). Class 1 and 2 integrons were detected in 180 (73.77%) and 5 (2.05%) strains, respectively, while class 3 integrons were absent. The amplicons ranged in size from 1.5 to 2.6 kb. A single band was obtained in 177 isolates and two bands were obtained in three isolates. Sequencing results revealed that the variant region of class 1 integron has four distinct gene cassettes (aadA1-catB8-aacA4, aacC1-OrfA-OrfB-aadA1, dfrA17-aadA5, aadA2-orfF-dfrA12), and the variant region of the class 2 integron has one gene cassette (dfrA1-sat2-aadA1-orfX) (Table 4).

**Table 4:** Characterization of integrons in *Acinetobacter* species

*intI* genes	Gene cassette array	No.(%) of isolates ^a^
1	aadA1-catB8-aacA4	169 (94.41)
	aacC1-OrfA-OrfB-aadA1	8 (4.47)
	dfrA17-aadA5	4 (2.23)
	aadA2-orfF-dfrA12	2 (1.12)
2	dfrA1-sat2-aadA1-orfX	5 (100)

^a^ No. of gene cassette positive isolates/no. of *intI* genes-positive isolates

### Eric-PCR

**Fig. 2:**
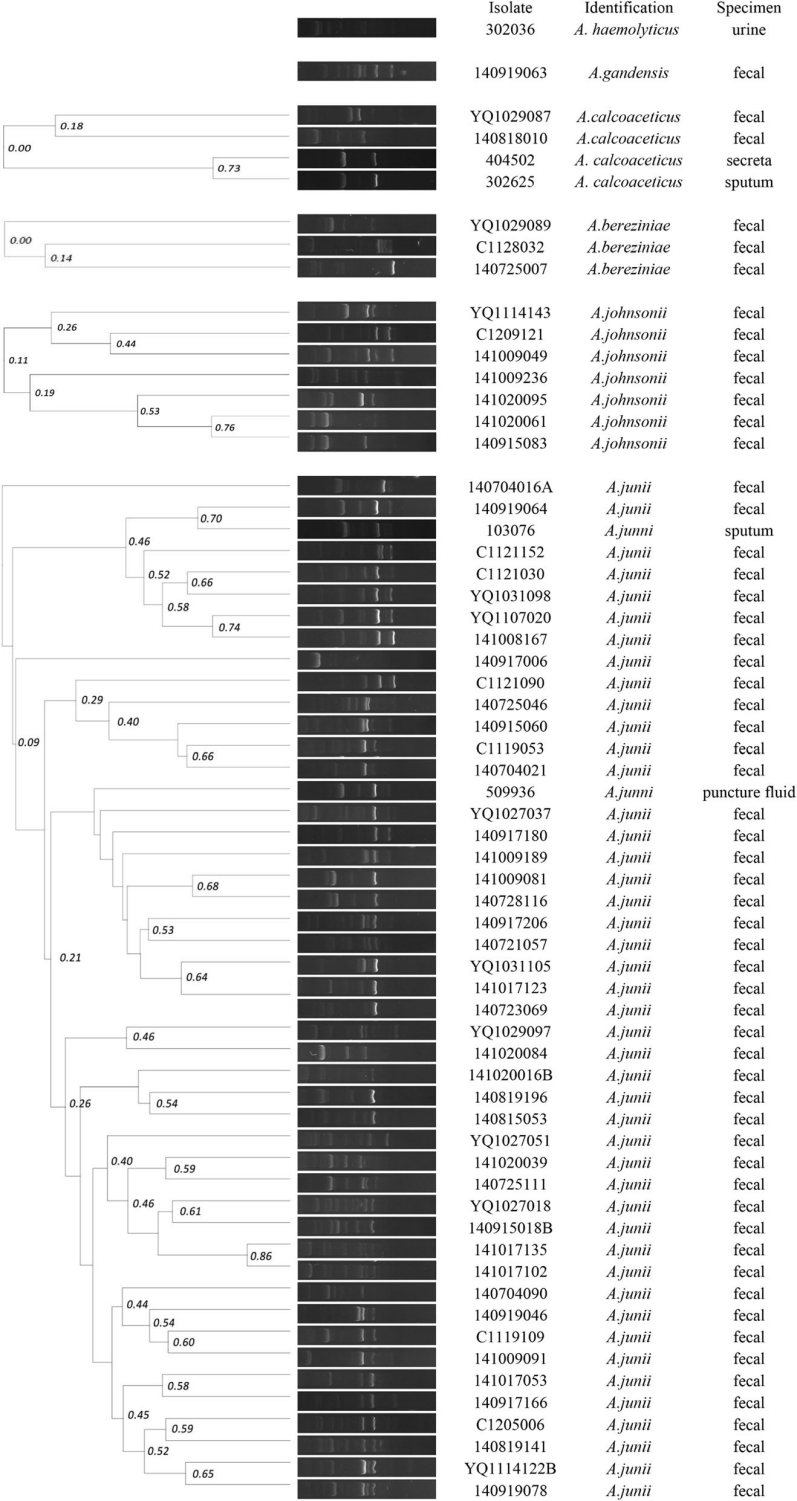
Dendrogram from ERIC-PCR analysis of 63 *Acinetobacter* isolates. In total, 62 distinct genotypes were identified based on 80% similarity. The scale bar indicates the similarity values. ERIC, enterobacterial repetitive intergenic consensus

Genotyping by ERIC-PCR demonstrated a high genetic diversity of *non-baumannii Acinetobacter* in different samples, and one was selected as a representative of the same band for dendrogram cluster analysis (Fig. 2). However, *A*. *baumannii* detected in clinical samples had greater than 90% similarity, suggesting they may have originated from the same clone.

## Discussion

The results of the carbapenem resistance gene sequencing showed that the *bla*_NDM_ gene was detected in 57 isolates and only present in the fecal survey samples. Fu et al. [40, 41] described that among *Acinetobacter* species, the *bla*_NDM_ gene is widely disseminated in China. It is possible that the spread of New Delhi metallo-beta-lactamase-1(NDM-1) carbapenemases may occur rapidly through *Acinetobacter spp.* because it may become markedly more difficult to eradicate [42] and has begun to steadily express NDM [43].

We showed that 98.90% of the *A*. *baumannii* isolates contained the *bla*_OXA-51-like_ gene. The results of Lowings et al. [44] and Correa et al. [45] indicated that 99 and 97.5% of the *A*. *baumannii* isolates contained this gene. Zhao et al. [46] showed 91.7% of the A. *baumannii* isolates contained this gene. Zander et al. [47] reported the identification of three *A*. *baumannii* isolates that did not harbor the predicted *bla*_OXA-51-like_ gene. Further study of the genomic environment of these isolates revealed changes in the *bla*_OXA-51-like_ genetic structure, resulting in variants *bla*_OXA-66_ or *bla*_OXA-78_, which were interrupted by the insertion sequence. An investigation conducted in 27 hospitals located in 14 European and Mediterranean countries during 2009–2011 showed that 277(67.4%) were positive for *bla*_OXA-23-like_ [48]. Correa et al. showed that 97.5% were positive for *bla*_*OXA-23-like*_ in Colombia (2008–2010) [45]. Moreover, 107(91.5%) isolates were positive for *bla*_OXA-23-like_ in the countries of the Gulf Cooperation Council [49], Carvalho et al. reported that 96 of 110 (87%) carried the *bla*_OXA-23-like_ gene in Rio de Janeiro [10]; however, in northern Croatia and Istria during 2009–2010, only 3.24% (6/185) of carbapenem-resistant *A*. *baumannii* isolates possessed the *bla*_OXA-23-like_ gene [50], while 25.7 and 28.3% were positive for the *bla*_OXA-24/40-like_ and *bla*_OXA-58-like_ genes, respectively, 152 (100%) isolates were negative for *bla*_OXA-23-like_ in Mexico [51], these data indicated that the prevalence or predominance of some OXA subgroups depends on geographic/regional variations.

In the present study, six (8.70%) *non-baumannii Acinetobacter* and one (0.55%) *A*. *baumannii* isolates were resistant to colistin. In 74 fecal survey samples, we detected that five colistin-resistant *A*. *junii* were positive for the *mcr-1* gene, and the *mcr* gene in *Acinetobacter* isolated from human samples had not been reported in the literature thus far. Moreover, all three *A*. *junii* (C1121030, C1121090, C1121152) contained *bla*_OXA-58-like_ and *bla*_NDM_, one of the three strains contained *bla*_NDM_, *bla*_VIM_, *bla*_OXA-58-like_, and *mcr-1*, while the remaining strains contained *bla*_NDM_, *bla*_IMP_, *bla*_OXA-58-like_, and *mcr-1*. This is the first report of more than two carbapenem resistance genes coexisting with the *mcr-1* gene in *Acinetobacter* species*.*

In the 74 fecal survey samples, 57 *bla*_NDM_ genes were detected; the *mcr-1* gene was detected in five isolates, and co-existed with carbapenem resistance genes. In 174 clinical infection samples, both the *bla*_NDM_ and *mcr-1* genes were absent, and no more than two carbapenemase genes co-existed. Comparison of clinical infection samples and fecal survey samples revealed that the detection rate of carbapenem resistance and *mcr-1* genes was higher in fecal survey samples, and the strains in which multiple MDR genes co-existed were all isolated from fecal samples, which may indicate that the intestinal tract is an important place for the transfer of bacterial resistance, reminding us of the importance to control nosocomial infections.

We observed a high mutation rate in the porin *carO* gene. The sequencing of *carO* genes revealed the presence of 11 distinct variants and 10 novel sequences. We analyzed the homology of *carO* gene in carbapenem-resistant Acinetobacter and carbapenem-sensitive reference strain ATCC 17978. The resulting nucleotide sequence and amino acid sequence exhibited 90 and 91% identity, respectively, with the *carO* sequence of ATCC 17978. When we compared these sequences with those available in different databanks (sequences from NCBI databanks), we identified a non-variable N-terminal domain (1–131) and two variable domains (132–162 and 200–238) by comparing amino acid sequences (Additional file 2: Figure S1), and two variable domains (397–480 and 588–677) by comparing nucleotide sequences (Additional file 3: Figure S2). We speculate that the structure and function of protein *CarO* will have considerable variation due to the presence of these two variable domains. The phenomenon of *CarO* participates in carbapenem influx has been reported, changes in the amino acid composition of *CarO* that could lead to altered porin may result in carbapenem resistance [17], although this will require further verification.

Many studies have described the harboring of the AdeABC efflux system in clinical isolates of *A*. *baumannii* [4]. Some studies have suggested that these genes are only present in MDR isolates while some studies have reported them in MDR and non-MDR isolates [52, 53]. Here, we report that the presence of *adeABC* and *adeIJK* in *A*. *baumannii* was 96.13 and 92.27%, but 12.77 and 2.13% in *A*. *junii*, respectively. Sirawit et al. [53] showed that the MDR phenotype in most *A*. *baumannii* isolates was associated with efflux pumps. AdeIJK expression was most common (97%), and 52% of the *A*. *baumannii* isolates simultaneously produced up to three RND-type efflux systems. Yoon et al. [54] detected the *AdeB* gene in 13 (92.86%) clinical isolates and *AdeG* and intrinsic *AdeJ* in all clinical isolates of *A*. *baumannii*. This is consistent with other studies [17], suggesting that efflux systems are likely species-specific. Overexpression of these pumps in response to antibiotic exposure leads to decreased susceptibility to the antibiotics [55].

*A*. *baumannii* was primarily isolated from clinical infection samples, and *non-baumannii Acinetobacter* was primarily isolated from fecal survey samples. ERIC-PCR demonstrated a high genetic diversity of *non-baumannii Acinetobacter* from different samples, but *A*. *baumannii* from clinical infection samples had greater than 90% similarity, suggesting that *A. baumannii* isolated from clinical infection samples may be from the same clone strain. However, all strains were isolated from samples collected from 2014 to 2015, thus indicating that there was no outbreak in the short term.

## Conclusion

The data demonstrated that carbapenem resistance genes were widely disseminated among *Acinetobacter* species in southern China, and multiple mechanisms contribute to carbapenem resistance. Colistin is the last line of defense to treat infection with carbapenem-resistant gram-negative bacteria, but the emergence of the *mcr* gene has brought greater challenges to clinical treatment. The appearance of notable clinical resistance genes such as *bla*_NDM_ and *mcr-1*, even in *non-baumannii Acinetobacter* gut colonizers, is important. According to the types and quantities of drug-resistant genes in *Acinetobacter* isolated from feces, the gut may be an important reservoir of resistant opportunistic bacteria.

## Additional files

Additional file 1: **Table S1.** a Sequence group and antibiotic susceptibility profile of all clinical infection samples carrying resistant genes. b Sequence group and antibiotic susceptibility profile of all clinical fecal survey samples carrying resistant genes. (DOCX 139 kb)

Additional file 2: **Figure S1.** *CarO* from *Acinetobacter* species. (a) Homology analysis of *CarO* amino acids sequences with ATCC 17978. (b) Alignment of *CarO* amino acids sequences , the *CarO* amino acids sequences of A6 strain and A592 strain are from NCBI databanks. (DOCX 15 kb)

Additional file 3: **Figure S2.** *carO* gene from *Acinetobacter* species. (a) Homology analysis of ten novel *carO* gene sequences with *carO* gene from ATCC 17978. (b) Alignment of *carO* gene sequences , the *carO* gene sequences of A6 strain and A592 strain are from NCBI databanks. (DOCX 124 kb)

## Data Availability

All data generated or analyzed in this study are available on request to the corresponding author.

## Abbreviations

*carO*: Carbapenem-associated outer membrane protein
ERIC: Enterobacterial repetitive intergenic consensus
MDR: Multidrug-resistant
OXA: Oxacillinase
RND: Resistance-nodulation-division
