# Supplementary material for: Molecular characteristics of carbapenem-resistant Acinetobacter spp. from clinical infection samples and fecal survey samples in Southern China
Source: BMC Infect Dis. 2019 Oct 28;19:900. doi: 10.1186/s12879-019-4423-3 (PMC6819553; doi:10.1186/s12879-019-4423-3)
Supplement: Supplementary file 1 — Table S1. a Sequence group and antibiotic susceptibility profile of all clinical infection samples carrying resistant genes. b Sequence group and antibiotic susceptibility profile of all clinical fecal survey samples carrying resistant genes. (DOCX 139 kb) [file 12879_2019_4423_MOESM1_ESM.docx]

**Table S1**

**a** Sequence group and antibiotic susceptibility profile of all clinical isolates carrying resistant genes

| Isolate | Identification | Specimen | MBLs | OXAs | *mcr-1* | *carO*^1^ | efflux system | | Mobile genetic elements | | | |  | MIC of antimicrobial(μg/ml)^3^ | | | | | | | | |
| --- | --- | --- | --- | --- | --- | --- | --- | --- | --- | --- | --- | --- | --- | --- | --- | --- | --- | --- | --- | --- | --- | --- |
|  |  |  |  |  |  |  |  |  | *IntI1* | *intI2* | Other^2^ | |  | AMK | GEN | IPM | MEM | CT | CIP | LVX | TET | SXT |
| 103286 | *A.baumannii* | sputum | - | 23,51 |  | *b* | *ABC* | *IJK* | 1 |  | *I*123 | *t*12 |  | >32 | >8 | >8 | >8 | **<=0.5** | >2 | >8 | >8 | >2/38 |
| 500929 | *A.baumannii* | puncture fluid | - | 23,51 |  | *b* | *ABC* | *IJK* | 1 |  | *I*123 | *t*1 |  | >32 | >8 | >8 | >8 | **<=0.5** | >2 | >8 | >8 | >2/38 |
| 302748A | *A.baumannii* | sputum | - | 23,51 |  | *b* | *ABC* | *IJK* | 1 |  | *I*123 | *t*12 |  | >32 | >8 | >8 | >8 | **<=0.5** | >2 | 8 | >8 | >2/38 |
| 302036 | *A. haemolyticus* | urine | - | 23 |  | *-* | *ABC* |  | 1 |  | *I*123 | *t*2 |  | **<=8** | >8 | >8 | 8 | **<=0.5** | >2 | >8 | >8 | >2/38 |
| 103442 | *A.baumannii* | sputum | - | 23,51 |  | *b* | *ABC* | *IJK* | 1 |  | *I*123 | *t*2 |  | >32 | >8 | >8 | >8 | **<=0.5** | >2 | 8 | >8 | >2/38 |
| 200518 | *A.baumannii* | sputum | - | 23,51 |  | *b* | *ABC* | *IJK* | 1 |  | *I*123 | *t*12 |  | >32 | >8 | >8 | >8 | **<=0.5** | >2 | >8 | >8 | >2/38 |
| 101802 | *A.baumannii* | sputum | - | 23,51 |  | *b* | *ABC* | *IJK* | 1 |  | *I*123 | *t*1 |  | >32 | >8 | >8 | >8 | **<=0.5** | >2 | >8 | >8 | >2/38 |
| 100838 | *A.baumannii* | sputum | - | 23,51 |  | *b* | *ABC* | *IJK* | - |  | *I*123 | *t*1 |  | >32 | >8 | >8 | >8 | **<=0.5** | >2 | >8 | >8 | >2/38 |
| 201476 | *A.baumannii* | sputum | - | 23,51 |  | *b* | *ABC* | *IJK* | - |  | *I*123 | *t*2 |  | >32 | >8 | >8 | >8 | **<=0.5** | >2 | >8 | >8 | >2/38 |
| 100588A | *A.baumannii* | puncture fluid | - | 23,51 |  | *a**^*^* | *ABC* | *IJK* | 1 |  | *I*123 | *t*12 |  | >32 | >8 | >8 | >8 | **<=0.5** | >2 | 8 | >8 | >2/38 |
| 100459 | *A.baumannii* | sputum | - | 23,51 |  | *b* | *ABC* | *IJK* | 1 |  | *I*123 |  |  | >32 | >8 | >8 | >8 | **<=0.5** | >2 | >8 | >8 | >2/38 |
| 302625 | *A**. calcoaceticus* | sputum | - | 23,51 |  | *b* | *ABC* |  | - |  | *I*123 | *t*2 |  | >32 | >8 | >8 | >8 | **<=0.5** | >2 | 8 | >8 | >2/38 |
| 300844 | *A.baumannii* | sputum | - | 23,51 |  | *b* | *ABC* | *IJK* | 1 |  | *I*123 | *t*1 |  | >32 | >8 | >8 | >8 | **<=0.5** | >2 | >8 | >8 | >2/38 |
| 500843 | *A.baumannii* | sputum | - | 23,51 |  | *b* | *ABC* | *IJK* | - |  | *I*123 | *t*1 |  | >32 | >8 | >8 | >8 | **<=0.5** | >2 | 8 | >8 | >2/38 |
| 404502 | *A. calcoaceticus* | secreta | - | 23,51 |  | *b* | *ABC* |  | - |  | *I*123 | *t*2 |  | >32 | >8 | >8 | >8 | **<=0.5** | >2 | 8 | >8 | >2/38 |
| 301645A | *A.baumannii* | sputum | - | 23,51 |  | *b* | *ABC* |  | 1 |  | *I*13 | *t*12 |  | >32 | >8 | >8 | >8 | **<=0.5** | >2 | 8 | >8 | **1/19** |
| 200574 | *A.baumannii* | puncture fluid | - | 23,51 |  | *b* |  | *IJK* | 1 |  | *I*123 | *t*2 |  | >32 | >8 | >8 | >8 | **<=0.5** | >2 | >8 | >8 | >2/38 |
| 100503A | *A.baumannii* | sputum | - | 23,51 |  | *b* | *ABC* | *IJK* | 1 |  | *I*123 | *t*1 |  | >32 | >8 | >8 | >8 | **<=0.5** | >2 | >8 | >8 | >2/38 |
| 101168A | *A.baumannii* | sputum | - | 23,51 |  | *b* | *ABC* | *IJK* | - |  | *I*123 | *t*2 |  | >32 | >8 | >8 | >8 | **<=0.5** | >2 | >8 | >8 | **2/38** |
| 302085 | *A.baumannii* | sputum | - | 23,51 |  | *b* | *ABC* | *IJK* | 1 |  | *I*123 |  |  | >32 | >8 | >8 | >8 | **<=0.5** | >2 | 8 | >8 | >2/38 |
| 502605 | *A.baumannii* | sputum | - | 23,51,58 |  | *b* | *ABC* | *IJK* | 1 |  | *I*123 | *t*1 |  | >32 | >8 | >8 | >8 | **<=0.5** | >2 | >8 | >8 | >2/38 |
| 102420A | *A.baumannii* | sputum | - | 23,51 |  | *-* | *ABC* | *IJK* | - |  | *I*123 | *t*2 |  | >32 | >8 | >8 | >8 | **<=0.5** | >2 | >8 | >8 | >2/38 |
| 500460 | *A.baumannii* | sputum | - | 23,51 |  | *b* | *ABC* | *IJK* | 1 |  | *I*123 | *t*2 |  | >32 | >8 | >8 | >8 | **<=0.5** | >2 | >8 | >8 | >2/38 |
| 502803 | *A.baumannii* | sputum | - | 23,51 |  | *b* | *ABC* | *IJK* | - |  | *I*123 | *t*12 |  | >32 | >8 | >8 | >8 | **<=0.5** | >2 | 8 | >8 | >2/38 |
| 300587 | *A.baumannii* | sputum | - | 23,51 |  | *b* | *ABC* | *IJK* | 1 |  | *I*123 | *t*12 |  | >32 | >8 | >8 | >8 | **<=0.5** | >2 | >8 | >8 | >2/38 |
| 404641 | *A.baumannii* | sputum | - | 23,51 |  | *b* | *ABC* | *IJK* | 1 |  | *I*123 | *t*2 |  | >32 | >8 | >8 | >8 | **<=0.5** | >2 | >8 | >8 | >2/38 |
| 300920 | *A.baumannii* | secreta | - | 23,51 |  | *b* | *ABC* | *IJK* | - |  | *I*123 | *t*12 |  | >32 | >8 | >8 | >8 | **<=0.5** | >2 | >8 | >8 | >2/38 |
| 101310 | *A.baumannii* | sputum | - | 23,51 |  | *b* | *ABC* | *IJK* | 1 |  | *I*123 | *t*1 |  | >32 | >8 | >8 | >8 | **<=0.5** | >2 | >8 | >8 | >2/38 |
| 301779 | *A.baumannii* | sputum | - | 23,51 |  | *b* | *ABC* |  | 1 |  | *I*123 | *t*2 |  | >32 | >8 | >8 | >8 | **<=0.5** | >2 | 8 | >8 | >2/38 |
| 101071B | *A.baumannii* | sputum | - | 23,51 |  | *b* | *ABC* | *IJK* | 1 |  | *I*123 | *t*1 |  | >32 | >8 | >8 | >8 | **<=0.5** | >2 | >8 | >8 | >2/38 |
| 101491A | *A.baumannii* | sputum | - | 23,51 |  | *b* | *ABC* | *IJK* | - |  | *I*123 | *t*1 |  | >32 | >8 | >8 | >8 | **<=0.5** | >2 | >8 | >8 | >2/38 |
| 500723 | *A.baumannii* | sputum | - | 23,51 |  | *b* | *ABC* | *IJK* | 1 |  | *I*123 | *t*12 |  | >32 | >8 | >8 | >8 | **<=0.5** | >2 | 8 | >8 | >2/38 |
| 103432 | *A.baumannii* | sputum | - | 23,51 |  | *b* | *ABC* | *IJK* | 1 |  | *I*123 | *t*12 |  | >32 | >8 | >8 | >8 | **<=0.5** | >2 | 8 | >8 | >2/38 |
| 100622 | *A.baumannii* | sputum | - | 23,51 |  | *b* | *ABC* | *IJK* | - |  | *I*123 | *t*2 |  | **<=8** | **<=2** | >8 | >8 | **<=0.5** | >2 | >8 | >8 | >2/38 |
| 500732 | *A.baumannii* | sputum | - | 23,51 |  | *b* | *ABC* | *IJK* | - |  | *I*123 | *t*2 |  | >32 | >8 | >8 | >8 | **<=0.5** | >2 | 8 | >8 | >2/38 |
| 500670 | *A.baumannii* | sputum | - | 23,51 |  | *b* | *ABC* | *IJK* | 1 |  | *I*123 | *t*12 |  | >32 | >8 | >8 | >8 | **<=0.5** | >2 | 8 | >8 | >2/38 |
| 302705A | *A.baumannii* | instrument | - | 23,51 |  | *b* | *ABC* |  | - |  | *I*123 | *t*1 |  | >32 | >8 | >8 | >8 | **<=0.5** | >2 | >8 | >8 | >2/38 |
| 103239 | *A.baumannii* | sputum | - | 23,51 |  | *b* | *ABC* | *IJK* | 1 |  | *I*123 |  |  | >32 | >8 | >8 | >8 | **<=0.5** | >2 | 8 | >8 | >2/38 |
| 200480A | *A.baumannii* | secreta | - | 23,51 |  | *b* | *ABC* | *IJK* | 1 |  | *I*123 | *t*1 |  | >32 | >8 | >8 | >8 | **<=0.5** | >2 | >8 | >8 | >2/38 |
| 500539 | *A.baumannii* | sputum | - | 23,51 |  | *b* | *ABC* | *IJK* | 1 |  | *I*123 | *t*2 |  | >32 | >8 | >8 | >8 | **<=0.5** | >2 | 8 | >8 | >2/38 |
| 500903 | *A.baumannii* | sputum | - | 23,51 |  | *b* | *ABC* | *IJK* | - |  | *I*123 | *t*2 |  | >32 | >8 | >8 | >8 | **<=0.5** | >2 | 8 | >8 | >2/38 |
| 200491 | *A.baumannii* | sputum | - | 23,51 |  | *b* | *ABC* | *IJK* | - |  | *I*23 | *t*2 |  | >32 | >8 | >8 | >8 | **<=0.5** | >2 | >8 | >8 | >2/38 |
| 102756A | *A.baumannii* | sputum | - | 23,51 |  | *b* | *ABC* | *IJK* | 1 |  | *I*13 | *t*2 |  | >32 | >8 | >8 | >8 | **<=0.5** | >2 | >8 | >8 | >2/38 |
| 103076 | *A.junni* | sputum | - | 23,51 |  | *-* | *-* | | - |  | *I*123 | *t*12 |  | >32 | >8 | >8 | >8 | **<=0.5** | >2 | >8 | >8 | >2/38 |
| 103332 | *A.baumannii* | sputum | - | 23,51 |  | *b* | *ABC* | *IJK* | 1 |  | *I*13 | *t*1 |  | >32 | >8 | >8 | >8 | **<=0.5** | >2 | >8 | >8 | >2/38 |
| 500546 | *A.baumannii* | sputum | - | 23,51 |  | *b* | *ABC* | *IJK* | 1 |  | *I*13 | *t*2 |  | >32 | >8 | >8 | >8 | **<=0.5** | >2 | >8 | >8 | >2/38 |
| 101172A | *A.baumannii* | sputum | - | 23,51 |  | *b* | *ABC* | *IJK* | - |  | *I*123 | *t*1 |  | >32 | >8 | >8 | >8 | **<=0.5** | >2 | >8 | >8 | >2/38 |
| 200515A | *A.baumannii* | sputum | - | 23,51 |  | *b* | *ABC* | *IJK* | 1 |  | *I*123 | t2 |  | >32 | >8 | >8 | >8 | **<=0.5** | >2 | >8 | >8 | >2/38 |
| 500603A | *A.baumannii* | sputum | - | 23,51 |  | *b* | *ABC* | *IJK* | 1 |  | *I*123 | *t*12 |  | >32 | >8 | >8 | >8 | **<=0.5** | >2 | 8 | >8 | >2/38 |
| 103278A | *A.baumannii* | sputum | - | 51,58 |  | *b* | *ABC* |  | 1 |  | *I*123 | *t*12 |  | >32 | >8 | >8 | >8 | **<=0.5** | >2 | 8 | >8 | >2/38 |
| 102208 | *A.baumannii* | sputum | - | 23,51 |  | *b* | *ABC* | *IJK* | 1 |  | *I*123 | *t*2 |  | >32 | >8 | >8 | >8 | **<=0.5** | >2 | >8 | >8 | >2/38 |
| 500736A | *A.baumannii* | sputum | - | 23,51 |  | *b* | *ABC* | *IJK* | - |  | *I*13 | *t*2 |  | >32 | >8 | >8 | >8 | **<=0.5** | >2 | 8 | >8 | >2/38 |
| 101384 | *A.baumannii* | sputum | - | 23,51 |  | *b* | *ABC* | *IJK* | 1 |  | *I*13 | *t*12 |  | >32 | >8 | >8 | >8 | **<=0.5** | >2 | >8 | >8 | >2/38 |
| 302737 | *A.baumannii* | urine | - | 23,51 |  | *b* | *ABC* | *IJK* | 1 |  | *I*13 | *t*2 |  | >32 | >8 | >8 | >8 | **<=0.5** | >2 | >8 | >8 | >2/38 |
| 102169 | *A.baumannii* | sputum | - | 23,51 |  | *b* | *ABC* | *IJK* | 1 |  | *I*13 | *t*2 |  | >32 | >8 | >8 | >8 | **<=0.5** | >2 | >8 | >8 | >2/38 |
| 101455 | *A.baumannii* | sputum | - | 23,51 |  | *b* | *ABC* | *IJK* | - |  | *I*13 | *t*1 |  | >32 | >8 | >8 | >8 | **<=0.5** | >2 | >8 | >8 | >2/38 |
| 500793A | *A.baumannii* | sputum | - | 23,51 |  | *b* | *ABC* |  | 1 |  | *I*13 | *t*12 |  | >32 | >8 | >8 | >8 | **<=0.5** | >2 | 8 | >8 | >2/38 |
| 103299 | *A.baumannii* | sputum | - | 23,51 |  | *b* | *ABC* | *IJK* | 1 |  | *I*13 | *t*2 |  | >32 | >8 | >8 | >8 | **<=0.5** | >2 | >8 | >8 | >2/38 |
| 200967 | *A.baumannii* | sputum | - | 23,51 |  | *b* | *ABC* | *IJK* | 1 |  | *I*123 | *t*1 |  | >32 | >8 | >8 | >8 | **<=0.5** | >2 | >8 | >8 | **1/19** |
| 300808 | *A.baumannii* | sputum | - | 23,51 |  | *b* | *ABC* | *IJK* | 1 |  | *I*123 | *t*2 |  | >32 | >8 | >8 | >8 | **<=0.5** | >2 | >8 | >8 | >2/38 |
| 301364A | *A.baumannii* | sputum | - | 23,51 |  | *b* |  | *IJK* | - |  | *I*123 | *t*12 |  | >32 | >8 | >8 | >8 | **<=0.5** | >2 | 8 | >8 | >2/38 |
| 302489 | *A.baumannii* | sputum | - | 23,51 |  | *b* | *ABC* | *IJK* | 1 |  | *I*13 | *t*12 |  | >32 | >8 | >8 | >8 | **<=0.5** | >2 | 8 | >8 | >2/38 |
| 301876 | *A.baumannii* | sputum | - | 23,51 |  | *b* | *ABC* | *IJK* | 1 |  | *I*13 |  |  | >32 | >8 | >8 | >8 | **<=0.5** | >2 | 8 | >8 | >2/38 |
| 404641 | *A.baumannii* | sputum | - | 23,51 |  | *b* | *ABC* | *IJK* | 1 |  | *I*13 | *t*1 |  | >32 | >8 | >8 | >8 | **<=0.5** | >2 | >8 | >8 | >2/38 |
| 404626 | *A.baumannii* | puncture fluid | - | 23,51 |  | *b* | *ABC* | *IJK* | 1 |  | *I*123 | *t*2 |  | >32 | >8 | >8 | >8 | **<=0.5** | >2 | 8 | >8 | >2/38 |
| 300427 | *A.baumannii* | sputum | - | 23,51 |  | *b* | *ABC* | *IJK* | - |  | *I*13 | *t*1 |  | >32 | >8 | >8 | >8 | **<=0.5** | >2 | >8 | >8 | >2/38 |
| 300316B | *A.baumannii* | sputum | - | 51 |  | *b* | *ABC* | *IJK* | 1 |  | *I*123 | *t*12 |  | >32 | >8 | >8 | >8 | **<=0.5** | >2 | >8 | >8 | >2/38 |
| 302079 | *A.baumannii* | sputum | - | 23,51 |  | *b* | *ABC* | *IJK* | - |  | *I*123 | *t*2 |  | >32 | >8 | >8 | >8 | **<=0.5** | >2 | 8 | >8 | **1/19** |
| 404697 | *A.baumannii* | sputum | - | 23,51 |  | *b* | *ABC* | *IJK* | - |  | *I*123 | *t*12 |  | >32 | >8 | >8 | >8 | **<=0.5** | >2 | >8 | >8 | >2/38 |
| 201937A | *A.baumannii* | sputum | IMP | 23,51 |  | *-* | *ABC* | *IJK* | 1 |  | *I*123 | *t*12 |  | >32 | >8 | >8 | >8 | **<=0.5** | >2 | >8 | >8 | >2/38 |
| 301273 | *A.baumannii* | sputum | - | 23,51 |  | *b* | *ABC* |  | 1 |  | *I*123 | *t*2 |  | >32 | >8 | >8 | >8 | **<=0.5** | >2 | >8 | >8 | >2/38 |
| 103399 | *A.baumannii* | sputum | - | 23,51 |  | *-* | *ABC* | *IJK* | 1 |  | *I*123 | *t*2 |  | >32 | >8 | >8 | >8 | **<=0.5** | >2 | 8 | >8 | >2/38 |
| 100628 | *A.baumannii* | sputum | - | 23,51 |  | *b* | *ABC* | *IJK* | 1 |  | *I*123 | *t*1 |  | >32 | >8 | >8 | >8 | **2** | >2 | >8 | >8 | >2/38 |
| 404655 | *A.baumannii* | instrument | - | 23,51 |  | *b* | *ABC* | *IJK* | - |  | *I*123 | *t*12 |  | >32 | >8 | >8 | >8 | **<=0.5** | >2 | 8 | >8 | >2/38 |
| 100529B | *A.baumannii* | secreta | - | 23,51 |  | *b* | *ABC* | *IJK* | 1 |  | *I*123 | *t*2 |  | >32 | >8 | >8 | >8 | **<=0.6** | >2 | >8 | >8 | >2/38 |
| 300045 | *A.baumannii* | secreta | - | 23,51 |  | *-* | *ABC* | *IJK* | 1 |  | *I*123 | *t*2 |  | >32 | >8 | >8 | >8 | **<=0.5** | >2 | >8 | >8 | >2/38 |
| 301366 | *A.baumannii* | sputum | - | 23,51 |  | *-* | *ABC* |  | 1 |  | *I*123 | *t*12 |  | >32 | >8 | >8 | >8 | **<=0.5** | >2 | >8 | >8 | >2/38 |
| 302853A | *A.baumannii* | secreta | - | 23,51 |  | *b* | *ABC* | *IJK* | - |  | *I*123 | *t*12 |  | >32 | >8 | >8 | >8 | **<=0.5** | >2 | 8 | >8 | >2/38 |
| 302047A | *A.baumannii* | sputum | - | 23,51 |  | *b* | *ABC* | *IJK* | 1 |  | *I*123 | *t*12 |  | >32 | >8 | >8 | >8 | **<=0.5** | >2 | 8 | >8 | **1/19** |
| 302833 | *A.baumannii* | sputum | - | 23,51 |  | *b* | *ABC* | *IJK* | - |  | *I*123 | *t*2 |  | >32 | >8 | >8 | >8 | **<=0.5** | >2 | >8 | >8 | >2/38 |
| 301267 | *A.baumannii* | sputum | - | 23,51 |  | *b* | *ABC* | *IJK* | 1 |  | *I*123 |  |  | >32 | >8 | >8 | >8 | **<=0.5** | >2 | >8 | >8 | **2/38** |
| 103011 | *A.baumannii* | sputum | - | 23,51 |  | *-* | *ABC* | *IJK* | - |  | *I*123 | *t*12 |  | >32 | >8 | >8 | >8 | **<=0.5** | >2 | >8 | >8 | >2/38 |
| 100171 | *A.baumannii* | sputum | - | 23,51 |  | *-* | *ABC* | *IJK* | 1 |  | *I*123 | *t*1 |  | >32 | >8 | >8 | >8 | **<=0.5** | >2 | >8 | >8 | >2/38 |
| 502536 | *A.baumannii* | sputum | - | 23,51 |  | *-* | *ABC* | *IJK* | - |  | *I*123 | *t*1 |  | >32 | >8 | >8 | >8 | **1** | >2 | >8 | >8 | >2/38 |
| 506313 | *A.baumannii* | sputum | - | 23,51 |  | *a* | *ABC* | *IJK* | - |  | *I*12 | *t*2 |  | **<=8** | >8 | >8 | >8 | **<=0.5** | **<=0.5** | **<=1** | >8 | >2/38 |
| 509121A | *A.baumannii* | sputum | - | 23,51 |  | *b* | *ABC* | *IJK* | - |  | *I*12 | *t*12 |  | >32 | >8 | >8 | >8 | **1** | >2 | >8 | >8 | **2/38** |
| 507217 | *A.baumannii* | sputum | - | 23,51 |  | *b* | *ABC* | *IJK* | 1 |  | *I*12 | *t*12 |  | >32 | >8 | >8 | >8 | **1** | >2 | >8 | >8 | >2/38 |
| 509519 | *A.baumannii* | sputum | - | 23,51 |  | *b* | *ABC* | *IJK* | - |  | *I*12 | *t*2 |  | >32 | >8 | >8 | >8 | **1** | >2 | >8 | >8 | >2/38 |
| 506563A | *A.baumannii* | sputum | - | 23,51 |  | *b* | *ABC* | *IJK* | 1 |  | *I*12 | *t*2 |  | >32 | >8 | >8 | >8 | **1** | >2 | >8 | >8 | **2/38** |
| 508859 | *A.baumannii* | sputum | - | 23,51 |  | *b* |  | *IJK* | - |  | *I*123 | *t*1 |  | >32 | >8 | >8 | >8 | **1** | >2 | >8 | >8 | >2/38 |
| 502816 | *A.baumannii* | sputum | - | 23,51 |  | *b* | *ABC* | *IJK* | 1 |  | *I*123 | *t*12 |  | >32 | >8 | >8 | >8 | **1** | >2 | >8 | >8 | >2/38 |
| 512831 | *A.baumannii* | sputum | - | 51 |  | *b* | *ABC* | *IJK* | 1 |  | *I*123 |  |  | >32 | >8 | >8 | >8 | **<=0.5** | >2 | >8 | >8 | >2/38 |
| 506561 | *A.baumannii* | sputum | - | 23,51 |  | *b* | *ABC* | *IJK* | - |  | *I*12 | *t*2 |  | >32 | >8 | >8 | >8 | **1** | >2 | >8 | >8 | >2/38 |
| 511608 | *A.baumannii* | sputum | - | 23,51 |  | *b* | *ABC* | *IJK* | 1 |  | *I*123 | *t*12 |  | >32 | >8 | >8 | >8 | **<=0.5** | >2 | >8 | >8 | >2/38 |
| 502957 | *A.baumannii* | instrument | - | 23,51 |  | *b* | *ABC* | *IJK* | 1 |  | *I*123 | *t*12 |  | >32 | >8 | >8 | >8 | **1** | >2 | >8 | >8 | **1/19** |
| 509122 | *A.baumannii* | sputum | - | 23,51 |  | *b* | *ABC* | *IJK* | - |  | *I*123 | *t*2 |  | >32 | >8 | >8 | >8 | **1** | >2 | >8 | >8 | >2/38 |
| 508023 | *A.baumannii* | sputum | - | 23,51 |  | *a* | *ABC* | *IJK* | - |  | *I*123 | *t*1 |  | **16** | >8 | >8 | >8 | **1** | **<=0.5** | **<=1** | >8 | >2/38 |
| 508996A | *A.baumannii* | instrument | - | 23,51 |  | *b* | *ABC* | *IJK* | 1 |  | *I*123 | *t*2 |  | >32 | >8 | >8 | >8 | **1** | >2 | >8 | >8 | >2/38 |
| 509159 | *A.baumannii* | sputum | - | 23,51 |  | *b* |  | *IJK* | 1 |  | *I*12 | *t*1 |  | >32 | >8 | >8 | >8 | **1** | >2 | 8 | >8 | >2/38 |
| 506075B | *A.baumannii* | sputum | - | 23,51 |  | *b* | *ABC* | *IJK* | 1 |  | *I*123 | *t*2 |  | >32 | >8 | >8 | >8 | >2 | >2 | >8 | >8 | >2/38 |
| 507373 | *A.baumannii* | sputum | - | 23,51 |  | *b* | *ABC* | *IJK* | - |  | *I*123 | *t*1 |  | >32 | >8 | >8 | >8 | **1** | >2 | >8 | >8 | >2/38 |
| 507055 | *A.baumannii* | sputum | - | 23,51 |  | *b* | *ABC* | *IJK* | 3 |  | *I*123 | *t*12 |  | >32 | >8 | >8 | >8 | **1** | >2 | >8 | >8 | >2/38 |
| 502732 | *A.baumannii* | sputum | - | 23,51 |  | *a* | *ABC* | *IJK* | 1 |  | *I*123 | *t*12 |  | **<=8** | >8 | >8 | >8 | **1** | **<=0.5** | **<=1** | >8 | >2/38 |
| 506454 | *A.baumannii* | sputum | - | 23,51 |  | *b* | *ABC* | *IJK* | - |  | *I*123 | *t*2 |  | >32 | >8 | >8 | >8 | **2** | >2 | >8 | >8 | >2/38 |
| 511328 | *A.baumannii* | sputum | - | 23,51,58 |  | *b* | *ABC* | *IJK* | 1 |  | *I*123 | *t*12 |  | >32 | >8 | >8 | >8 | **<=0.5** | >2 | >8 | >8 | >2/38 |
| 511607A | *A.baumannii* | sputum | - | 23,51 |  | *b* | *ABC* | *IJK* | - |  | *I*123 | *t*12 |  | >32 | >8 | >8 | >8 | **<=0.5** | >2 | >8 | >8 | **2/38** |
| 502992 | *A.baumannii* | sputum | - | 23,51 |  | *b* | *ABC* | *IJK* | 1 |  | *I*123 | *t*1 |  | >32 | >8 | >8 | >8 | **1** | >2 | >8 | >8 | >2/38 |
| 511347 | *A.baumannii* | sputum | - | 51 |  | *b* | *ABC* | *IJK* | 2 |  | *I*123 | *t*12 |  | >32 | >8 | >8 | >8 | **<=0.5** | >2 | >8 | >8 | >2/38 |
| 507946 | *A.baumannii* | sputum | - | 23,51 |  | *a* | *ABC* | *IJK* | 1 |  | *I*123 | *t*1 |  | **<=8** | >8 | >8 | >8 | **1** | **<=0.5** | **<=1** | **<=2** | >2/38 |
| 513007 | *A.baumannii* | sputum | - | 23,51 |  | *a^*^* | *ABC* | *IJK* | 1 |  | *I*123 | *t*12 |  | **<=8** | >8 | >8 | >8 | **<=0.5** | **<=0.5** | **<=1** | >8 | >2/38 |
| 506086B | *A.baumannii* | sputum | - | 23,51 |  | *a* | *ABC* |  | 4 |  | *I*123 | *t*1 |  | >32 | >8 | >8 | >8 | **1** | >2 | >8 | >8 | >2/38 |
| 511171 | *A.baumannii* | sputum | - | 23,51 |  | *b* | *ABC* | *IJK* | - |  | *I*123 | *t*12 |  | >32 | >8 | >8 | >8 | **1** | >2 | >8 | >8 | >2/38 |
| 506894B | *A.baumannii* | sputum | - | 23,51 |  | *b* | *ABC* | *IJK* | 1 |  | *I*123 | *t*12 |  | >32 | >8 | >8 | >8 | **1** | >2 | >8 | >8 | >2/38 |
| 503272 | *A.baumannii* | sputum | - | 23,51 |  | *b* | *ABC* | *IJK* | 1 |  | *I*123 | *t*12 |  | >32 | >8 | >8 | >8 | **1** | >2 | >8 | >8 | >2/38 |
| 511722C | *A.baumannii* | sputum | - | 23,51 |  | *b* | *ABC* | *IJK* | 1 |  | *I*123 | *t*12 |  | >32 | >8 | >8 | >8 | **<=0.5** | >2 | >8 | >8 | >2/38 |
| 506408 | *A.baumannii* | sputum | - | 23,51 |  | *b* | *ABC* | *IJK* | 1 |  | *I*123 | *t*1 |  | >32 | >8 | >8 | >8 | **1** | >2 | >8 | >8 | >2/38 |
| 510697 | *A.baumannii* | sputum | - | 23,51 |  | *a* | *ABC* | *IJK* | 1 |  | *I*123 | *t*12 |  | **<=8** | >8 | >8 | >8 | **1** | **<=0.5** | **<=1** | >8 | >2/38 |
| 509936 | *A.junni* | puncture fluid | - | 23,51 |  | *b* | *-* | | 1  3 |  | *I*123 |  |  | >32 | >8 | >8 | >8 | **1** | >2 | >8 | >8 | >2/38 |
| 512170 | *A.baumannii* | sputum | - | 23,51 |  | *a* | *ABC* | *IJK* | 1 |  | *I*123 | *t*1 |  | **<=8** | >8 | >8 | >8 | **<=0.5** | **<=0.5** | **<=1** | >8 | >2/38 |
| 511179B | *A.baumannii* | sputum | - | 23,51 |  | *a* | *ABC* | *IJK* | 1 |  | *I*123 | *t*12 |  | **<=8** | >8 | >8 | >8 | **1** | **<=0.5** | **<=1** | >8 | >2/38 |
| 509878 | *A.baumannii* | sputum | - | 23,51 |  | *a* | *ABC* | *IJK* | - |  | *I*123 | *t*12 |  | **<=8** | >8 | >8 | >8 | **1** | **<=0.5** | **<=1** | >8 | >2/38 |
| 511228A | *A.baumannii* | sputum | - | 23,51 |  | *b* |  | *IJK* | 1 |  | *I*123 | *t*2 |  | >32 | >8 | >8 | >8 | **<=0.5** | >2 | >8 | >8 | **1/19** |
| 505945 | *A.baumannii* | sputum | - | 23,51 |  | *b* | *ABC* | *IJK* | 1 |  | *I*123 | *t*12 |  | 32 | >8 | >8 | >8 | **2** | >2 | >8 | >8 | >2/38 |
| 508209 | *A.baumannii* | sputum | - | 23,51 |  | *b* | *ABC* | *IJK* | - |  | *I*123 | *t*1 |  | **16** | >8 | >8 | >8 | **1** | **1** | **<=1** | >8 | >2/38 |
| 506455 | *A.baumannii* | sputum | - | 23,51 |  | *a* | *ABC* | *IJK* | 1 |  | *I*123 | *t*2 |  | **16** | >8 | >8 | >8 | **1** | **<=0.5** | **<=1** | >8 | >2/38 |
| 508927A | *A.baumannii* | sputum | - | 23,51 |  | *a* | *ABC* | *IJK* | 1 |  | *I*123 |  |  | **<=8** | >8 | >8 | >8 | **1** | **<=0.5** | **<=1** | >8 | >2/38 |
| 506086B | *A.baumannii* | sputum | - | 23,51 |  | *b* | *ABC* | *IJK* | 2 |  | *I*123 | *t*12 |  | >32 | >8 | >8 | >8 | **1** | >2 | >8 | >8 | >2/38 |
| 506591B | *A.baumannii* | secreta | - | 23,51 |  | *b* | *ABC* | *IJK* | 1 |  | *I*123 | *t*12 |  | >32 | >8 | >8 | >8 | **1** | >2 | >8 | >8 | **2/38** |
| 509161 | *A.baumannii* | sputum | - | 23,51 |  | *b* | *ABC* | *IJK* | 1 |  | *I*123 | *t*1 |  | >32 | >8 | >8 | >8 | **<=0.5** | >2 | >8 | >8 | >2/38 |
| 506462B | *A.baumannii* | sputum | - | 23,51 |  | *a* | *ABC* | *IJK* | 1 |  | *I*123 | *t*2 |  | **16** | >8 | >8 | >8 | **1** | **<=0.5** | **<=1** | >8 | >2/38 |
| 506188 | *A.baumannii* | sputum | - | 23,51 |  | *b* | *ABC* | *IJK* | 1 |  | *I*23 | *t*12 |  | >32 | >8 | >8 | >8 | **1** | >2 | >8 | >8 | >2/38 |
| 509775 | *A.baumannii* | secreta | - | 23,51 |  | *b* | *ABC* | *IJK* | - |  | *I*123 | *t*12 |  | >32 | >8 | >8 | >8 | **<=0.5** | >2 | >8 | >8 | >2/38 |
| 503536 | *A.baumannii* | sputum | - | 23,51 |  | *b* | *ABC* | *IJK* | 1 |  | *I*123 | *t*12 |  | >32 | >8 | >8 | >8 | **1** | >2 | >8 | >8 | **2/38** |
| 511177 | *A.baumannii* | sputum | - | 51,58 |  | *b* | *ABC* | *IJK* | - |  | *I*123 | *t*2 |  | >32 | >8 | >8 | >8 | **1** | >2 | >8 | >8 | >2/38 |
| 503026A | *A.baumannii* | sputum | - | 23,51 |  | *b* | *ABC* | *IJK* | 1 |  | *I*123 | *t*1 |  | >32 | >8 | >8 | >8 | **1** | >2 | >8 | >8 | >2/38 |
| 511326 | *A.baumannii* | sputum | - | 23,51 |  | *b* | *ABC* | *IJK* | 1 |  | *I*123 | *t*12 |  | >32 | >8 | >8 | >8 | **<=0.5** | >2 | >8 | >8 | >2/38 |
| 511229B | *A.baumannii* | sputum | - | 23,51 |  | *b* | *ABC* |  | 1 |  | *I*12 | *t*1 |  | >32 | >8 | >8 | >8 | **<=0.5** | >2 | >8 | >8 | >2/38 |
| 503238 | *A.baumannii* | sputum | - | 23,51 |  | *b* | *ABC* | *IJK* | 1 |  | *I*123 | *t*2 |  | >32 | >8 | >8 | >8 | **<=0.5** | >2 | >8 | >8 | >2/38 |
| 506461 | *A.baumannii* | sputum | - | 23,51 |  | *a* | *ABC* | *IJK* | 1 |  | *I*123 | *t*12 |  | **16** | >8 | >8 | >8 | **1** | **<=0.5** | **<=1** | >8 | >2/38 |
| 506149 | *A.baumannii* | sputum | - | 23,51 |  | *b* | *ABC* | *IJK* | 1 |  | *I*123 | *t*12 |  | **16** | >8 | >8 | >8 | **1** | >2 | >8 | >8 | >2/38 |
| 506587 | *A.baumannii* | instrument | - | 51,58 |  | *b* | *ABC* | *IJK* | 1 |  | *I*123 | *t*12 |  | >32 | >8 | >8 | >8 | **<=0.5** | >2 | >8 | >8 | >2/38 |
| 502823A | *A.baumannii* | sputum | - | 23,51 |  | *b* | *ABC* | *IJK* | 1  2 |  | *I*123 | *t*1 |  | >32 | >8 | >8 | >8 | **1** | >2 | >8 | >8 | >2/38 |
| 512065A | *A.baumannii* | puncture fluid | - | 23,51 |  | *b* | *ABC* | *IJK* | 1 |  | *I*123 | *t*1 |  | >32 | >8 | >8 | >8 | **<=0.5** | >2 | >8 | >8 | >2/38 |
| 505957 | *A.baumannii* | sputum | - | 23,51 |  | *b* | *ABC* | *IJK* | 1 |  | *I*123 | *t*12 |  | **<=8** | >8 | >8 | >8 | **1** | >2 | >8 | >8 | >2/38 |
| 507249 | *A.baumannii* | sputum | - | 23,51 |  | *a* | *ABC* | *IJK* | 1 |  | *I*23 | *t*12 |  | **16** | >8 | >8 | >8 | **1** | **<=0.5** | **<=1** | >8 | >2/38 |
| 30842 | *A.baumannii* | blood | - | 23,51,58 |  | *b* | *ABC* | *IJK* | 1 |  | *I*123 | *t*12 |  | >32 | >8 | >8 | 8I | **1** | >2 | >8 | >8 | >2/38 |
| 510705 | *A.baumannii* | sputum | - | 23,51 |  | *k* | *ABC* | *IJK* | 1 |  | *I*23 | *t*12 |  | >32 | >8 | >8 | >8 | **1** | >2 | >8 | >8 | >2/38 |
| 503608B | *A.baumannii* | sputum | - | 23,51 |  | *b* | *ABC* | *IJK* | 1 |  | *I*12 | *t*12 |  | >32 | >8 | >8 | >8 | **<=0.5** | >2 | >8 | >8 | >2/38 |
| 508055 | *A.baumannii* | sputum | - | 23,51 |  | *b* | *ABC* | *IJK* | - |  | *I*123 | *t*12 |  | >32 | >8 | >8 | >8 | **1** | >2 | >8 | >8 | >2/38 |
| 507323 | *A.baumannii* | sputum | - | 23,51 |  | *b* | *ABC* |  | 1 |  | *I*123 | *t*1 |  | >32 | >8 | >8 | >8 | **1** | >2 | >8 | >8 | >2/38 |
| 509875 | *A.baumannii* | sputum | - | 23,51 |  | *b* | *ABC* | *IJK* | 1 |  | *I*123 | *t*12 |  | >32 | >8 | 8 | >8 | **1** | >2 | >8 | >8 | >2/38 |
| 503256 | *A.baumannii* | sputum | - | 23,51 |  | *-* | *ABC* | *IJK* | - |  | *I*123 | *t*12 |  | >32 | >8 | >8 | >8 | **<=0.5** | >2 | >8 | >8 | >2/38 |
| 513268 | *A.baumannii* | sputum | - | 23,51 |  | *-* | *ABC* | *IJK* | 1 |  | *I*123 | *t*12 |  | >32 | >8 | >8 | >8 | **<=0.5** | >2 | >8 | >8 | >2/38 |
| 503061 | *A.baumannii* | sputum | - | 23,51 |  | *b* | *ABC* | *IJK* | 1 |  | *I*123 | *t*12 |  | >32 | >8 | >8 | >8 | **1** | >2 | >8 | >8 | **2/38** |
| 506016 | *A.baumannii* | sputum | - | 23,51 |  | *-* | *ABC* | *IJK* | 1 |  | *I*123 | *t*12 |  | >32 | >8 | >8 | >8 | **<=0.5** | >2 | >8 | >8 | >2/38 |
| 513510 | *A.baumannii* | sputum | - | 23,51 |  | *-* | *ABC* | *IJK* | 1 |  | *I*123 | *t*12 |  | >32 | >8 | >8 | >8 | **<=0.5** | >2 | >8 | >8 | >2/38 |
| 509355 | *A.baumannii* | sputum | - | 23,51 |  | *b* | *ABC* | *IJK* | 1 |  | *I*123 | *t*12 |  | >32 | >8 | >8 | >8 | **1** | >2 | >8 | >8 | >2/38 |
| 507078B | *A.baumannii* | sputum | - | 23,51 |  | *-* | *ABC* | *IJK* | 1  2 |  | *I*12 | *t*12 |  | >32 | >8 | >8 | >8 | **1** | >2 | >8 | >8 | >2/38 |
| 513307 | *A.baumannii* | puncture fluid | - | 23,51 |  | *-* | *ABC* |  | 1 |  | *I*123 | *t*1 |  | >32 | >8 | >8 | >8 | **<=0.5** | >2 | >8 | >8 | >2/38 |
| 505937 | *A.baumannii* | sputum | - | 23,51 |  | *-* | *ABC* | *IJK* | 1 |  | *I*123 | *t*12 |  | >32 | >8 | >8 | >8 | **1** | >2 | >8 | >8 | **1/19** |
| 507814 | *A.baumannii* | sputum | - | 23,51 |  | *-* | *ABC* | *IJK* | 1 |  | *I*123 | *t*12 |  | >32 | >8 | >8 | >8 | **1** | >2 | >8 | >8 | **2/38** |
| 506896 | *A.baumannii* | sputum | - | 23,51 |  | *-* | *ABC* | *IJK* | 1 |  | *I*23 | *t*2 |  | **16** | >8 | >8 | >8 | **1** | **<=0.5** | **<=1** | >8 | >2/38 |
| 507688 | *A.baumannii* | sputum | - | 23,51 |  | *-* | *ABC* | *IJK* | - |  | *I*23 | *t*1 |  | >32 | >8 | >8 | >8 | **1** | >2 | >8 | >8 | >2/38 |
| 508351A | *A.baumannii* | sputum | - | 23,51 |  | *-* |  | *IJK* | 1 |  | *I*123 | *t*12 |  | >32 | >8 | >8 | >8 | **1** | >2 | >8 | >8 | >2/38 |
| 512702 | *A.baumannii* | sputum | - | 23,51 |  | *-* | *ABC* | *IJK* | 1 |  | *I*123 | *t*12 |  | >32 | >8 | >8 | >8 | **<=0.5** | >2 | 8 | >8 | >2/38 |
| 512812 | *A.baumannii* | puncture fluid | - | 23,51 |  | *b* | *ABC* | *IJK* | 1 |  | *I*12 | *t*12 |  | >32 | >8 | >8 | >8 | **<=0.5** | >2 | >8 | >8 | >2/38 |
| 513257A | *A.baumannii* | sputum | - | 23,51 |  | *b* | *ABC* | *IJK* | 1 |  | *I*123 | *t*2 |  | >32 | >8 | >8 | >8 | **<=0.5** | >2 | >8 | >8 | >2/38 |
| 512433 | *A.baumannii* | sputum | - | 23,51 |  | *-* | *ABC* | *IJK* | 1 |  | *I*123 | *t*12 |  | **<=8** | >8 | >8 | >8 | **<=0.5** | **<=0.5** | **<=1** | >8 | >2/38 |
| 512484 | *A.baumannii* | sputum | - | 23,51 |  | *b* | *ABC* | *IJK* | 1 |  | *I*123 | *t*2 |  | >32 | >8 | >8 | >8 | **<=0.5** | >2 | >8 | >8 | >2/38 |
| 513385 | *A.baumannii* | sputum | - | 23,51 |  | *b* | *ABC* | *IJK* | 2 |  | *I*12 | *t*2 |  | >32 | >8 | >8 | >8 | **<=0.5** | >2 | >8 | >8 | >2/38 |

**b** Sequence group and antibiotic susceptibility profile of all non-clinical fecal isolates carrying resistant genes

| Isolate | Identification | Specimen | MBLs | OXAs | *mcr-1* | *carO* | efflux system | | Mobile genetic elements | | | |  | MIC of antimicrobial(μg/ml) | | | | | | | | |
| --- | --- | --- | --- | --- | --- | --- | --- | --- | --- | --- | --- | --- | --- | --- | --- | --- | --- | --- | --- | --- | --- | --- |
|  |  |  |  |  |  |  |  |  | *intI1* | *intI2* | other | |  | AMK | GEN | IPM | MEM | CT | CIP | LVX | TET | SXT |
| 140604269 | *A.baumannii* | fecal | - | 23,51 |  | *b* | *ABC* | *IJK* | 1 |  | *I*13 | *t*1 |  | >32 | >8 | >8 | >8 | **1** | >2 | 8 | >8 | >2/38 |
| 140605460 | *A.baumannii* | fecal | - | 23,51 |  | *b* | *ABC* | *IJK* | 1 |  | *I*123 | *t*12 |  | >32 | >8 | >8 | >8 | **<=0.5** | >2 | >8 | >8 | >2/38 |
| 140627012 | *A. baumannii* | fecal | - | 23,51 |  | *b* | *ABC* |  | 1 |  | *I*123 |  |  | **<=8** | >8 | >8 | >8 | **1** | >2 | >8 | >8 | >2/38 |
| 140704021 | *A.junii* | fecal | IMP | - |  | - | *ABC* |  | - | 5 | *I*123 | *t*2 |  | >32 | >8 | >8 | >8 | **<=0.5** | >2 | >8 | 8 | >2/38 |
| 140704090 | *A.junii* | fecal | NDM | 58 |  | - | *-* | | 1 |  | *I*123 |  |  | **16** | >8 | >8 | >8 | **1** | >2 | >8 | 8 | >2/38 |
| 140723069 | *A.junii* | fecal | NDM | 58 |  | - | *-* | | 1 |  | *I*13 | *t*2 |  | **<=8** | >8 | >8 | >8 | **<=0.5** | >2 | 8 | 8 | >2/38 |
| 140723077 | *A.baumannii* | fecal | - | 24,51 |  | *c* | *ABC* | *IJK* | 1 |  | *I*123 | *t*2 |  | **<=8** | **<=2** | >8 | >8 | **<=0.5** | **<=0.5** | **<=1** | **<=2** | >2/38 |
| 140725007 | *A.bereziniae* | fecal | NDM | - |  | *-* | *ABC* | *IJK* | 1 |  | *I*123 | *t*12 |  | **16** | >8 | >8 | >8 | **<=0.5** | >2 | 8 | **<=2** | >2/38 |
| 140725046 | *A.junii* | fecal | NDM | 58 |  | - | *-* | | - |  | *I*123 | *t*1 |  | **<=8** | >8 | >8 | >8 | **<=0.5** | >2 | **2** | 8 | >2/38 |
| 140725111 | *A.junii* | fecal | NDM | 58 |  | - | - | | 1 |  | *I*123 | *t*2 |  | **<=8** | >8 | >8 | 8 | **1** | >2 | 4 | **4** | >2/38 |
| 140728116 | *A.junii* | fecal | NDM | - |  | - | *-* | | 1 |  | *I*123 | *t*1 |  | **<=8** | >8 | >8 | >8 | **<=0.5** | >2 | 4 | >8 | >2/38 |
| 140728137 | *A.baumannii* | fecal | - | 24,51 |  | *d* |  | *IJK* | 1 |  | *I*123 | *t*1 |  | **<=8** | **<=2** | >8 | 8 | **<=0.5** | **<=0.5** | **<=1** | **<=2** | **<=0.5/9.5** |
| 140815053 | *A.junii* | fecal | NDM | 58 |  | - | *ABC* |  | 1 |  | *I*123 | *t*2 |  | **<=8** | >8 | >8 | >8 | **<=0.5** | >2 | 8 | >8 | >2/38 |
| 140704006A | *A.baumannii* | fecal | - | 24,51 |  | *f* | *ABC* | *IJK* | - |  | *I*13 | *t*1 |  | **<=8** | **<=2** | 8 | 8 | **<=0.5** | **<=0.5** | **<=1** | **<=2** | **<=0.5/9.5** |
| 140704016A | *A.junii* | fecal | NDM | - |  | - | - | | - |  | *I*123 | *t*2 |  | **<=8** | >8 | >8 | >8 | **<=0.5** | >2 | 4 | **4** | >2/38 |
| 140721057 | *A.junii* | fecal | VIM, NDM | 58 |  | - | *-* | | 2 |  | *I*123 |  |  | >32 | >8 | >8 | >8 | **<=0.5** | >2 | >8 | **4** | >2/38 |
| 140725001A | *A.baumannii* | fecal | - | 24,51 |  | *c* | *ABC* | *IJK* | 1 |  | *I*123 | *t*12 |  | **<=8** | **<=2** | >8 | >8 | **<=0.5** | **<=0.5** | **<=1** | **<=2** | **<=0.5/9.5** |
| 140818010 | *A.calcoaceticus* | fecal | NDM | 58 |  | *j* | *ABC* |  | 2 |  | *I*123 | *t*1 |  | >32 | >8 | >8 | >8 | **<=0.5** | **<=0.5** | **<=1** | 8 | >2/38 |
| 140818091 | *A.baumannii* | fecal | VIM | 23,51 |  | *b* | *ABC* | *IJK* | 1 | 5 | *I*123 | *t*12 |  | >32 | >8 | >8 | >8 | **1** | >2 | >8 | >8 | >2/38 |
| 140819141 | *A.junii* | fecal | NDM | - |  | - | *-* | | 1 |  | *I*123 | *t*12 |  | **<=8** | **<=2** | >8 | 8 | **<=0.5** | >2 | **2** | **4** | **1/19** |
| 140819196 | *A.junii* | fecal | NDM | - |  | - | *-* | | - |  | *I*123 | *t*2 |  | **<=8** | **<=2** | >8 | >8 | **<=0.5** | >2 | 4 | 8 | >2/38 |
| 140915018B | *A.junii* | fecal | NDM | - | + | - | *-* | | 1 |  | *I*123 |  |  | **<=8** | >8 | >8 | 8 | >2 | >2 | **2** | >8 | **1/19** |
| 140915060 | *A.junii* | fecal | NDM | - |  | - | *-* | | 1 |  | *I*123 | *t*12 |  | **<=8** | **<=2** | >8 | 8 | **<=0.5** | >2 | 4 | **4** | >2/38 |
| 140915083 | *A.johnsonii* | fecal | NDM | 58 |  | - |  | *IJK* | 1 |  | *I*123 | *t*2 |  | **<=8** | >8 | >8 | >8 | **<=0.5** | >2 | 8 | **4** | >2/38 |
| 140917006 | *A.junii* | fecal | - | 58 |  | - | - | | 1 |  | *I*123 | *t*1 |  | **<=8** | >8 | >8 | >8 | **<=0.5** | >2 | 4 | >8 | >2/38 |
| 140917166 | *A.junii* | fecal | NDM | 58 |  | - | *-* | | - |  | *I*13 | *t*2 |  | **<=8** | >8 | >8 | >8 | **1** | >2 | 8 | >8 | >2/38 |
| 140917180 | *A.junii* | fecal | NDM | - |  | - | *ABC* |  | 1 |  | *I*12 | *t*1 |  | **<=8** | 8 | >8 | 8 | **<=0.5** | >2 | **2** | **<=2** | >2/38 |
| 140917184 | *A.baumannii* | fecal | VIM, NDM | 23,51 |  | *b* | *ABC* |  | 1 |  | *I*13 | *t*2 |  | **<=8** | >8 | >8 | >8 | **1** | >2 | >8 | >8 | >2/38 |
| 140917206 | *A.junii* | fecal | NDM | 58 |  | - | *ABC* |  | - |  | *I*123 | *t*12 |  | **<=8** | **<=2** | >8 | >8 | **<=0.5** | 2 | **<=1** | **<=2** | **1/19** |
| 140919046 | *A.junii* | fecal | NDM | - |  | - | - | | 1 |  | *I*123 | *t*2 |  | >32 | >8 | >8 | >8 | **<=0.5** | >2 | 8 | 8 | **1/19** |
| 140919063 | *A.gandensis* | fecal | NDM | 58 |  | - | - | | 1 |  | *I*123 | *t*1 |  | **<=8** | >8 | >8 | >8 | **<=0.5** | >2 | 8 | 8 | >2/38 |
| 140919064 | *A.junii* | fecal | NDM | 58 |  | - | *-* | | - |  | *I*123 | *t*2 |  | **<=8** | >8 | >8 | >8 | **1** | **<=0.5** | **<=1** | **4** | >2/38 |
| 140919078 | *A.junii* | fecal | NDM | - |  | - | - | | - |  | *I*13 | *t*1 |  | **<=8** | **<=2** | >8 | >8 | **1** | >2 | **2** | **4** | **<=0.5/9.5** |
| 141008167 | *A.junii* | fecal | - | 58 |  | - | *ABC* |  | 1 |  | *I*123 | *t*1 |  | **16** | >8 | >8 | >8 | **<=0.5** | >2 | >8 | >8 | >2/38 |
| 141009049 | *A.johnsonii* | fecal | NDM | - |  | - |  | *IJK* | - |  | *I*123 | *t*12 |  | **<=8** | **<=2** | >8 | >8 | **<=0.5** | **<=0.5** | **<=1** | **<=2** | **1/19** |
| 141009081 | *A.junii* | fecal | NDM | - |  | - | *-* | | 3 |  | *I*123 | *t*1 |  | **<=8** | >8 | >8 | >8 | **<=0.5** | >2 | 4 | 8 | >2/38 |
| 141009091 | *A.junii* | fecal | NDM | - |  | - | - | | 1 |  | *I*13 | *t*12 |  | **<=8** | >8 | >8 | >8 | **<=0.5** | >2 | 4 | **4** | >2/38 |
| 141009189 | *A.junii* | fecal | NDM | - |  | - | - | | 1 |  | *I*123 |  |  | **<=8** | >8 | >8 | >8 | **<=0.5** | >2 | 4 | >8 | >2/38 |
| 141009236 | *A.johnsonii* | fecal | NDM | 23 |  | *-* |  | *IJK* | 1 |  | *I*123 |  |  | **<=8** | **<=2** | >8 | >8 | **<=0.5** | >2 | **2** | **4** | **<=0.5/9.5** |
| 141009296 | *A.baumannii* | fecal | NDM | - |  | *h* | *ABC* | *IJK* | - |  | *I*123 | *t*2 |  | **16** | >8 | >8 | >8 | **<=0.5** | **1** | **<=1** | **4** | >2/38 |
| 141013051A | *A.baumannii* | fecal | - | - |  | - | *ABC* | *IJK* | - |  | *I*13 | *t*1 |  | >32 | >8 | >8 | >8 | **<=0.5** | >2 | >8 | >8 | >2/38 |
| 141017053 | *A.junii* | fecal | NDM | - |  | - | - | | 1 |  | *I*123 | *t*2 |  | **<=8** | >8 | 8 | 8 | **<=0.5** | 2 | **2** | **<=2** | >2/38 |
| 141017102 | *A.junii* | fecal | NDM | - |  | - | - | | 1 |  | *I*123 | *t*12 |  | **<=8** | >8 | >8 | 8 | **<=0.5** | >2 | **2** | **4** | **1/19** |
| 141017123 | *A.junii* | fecal | NDM | - |  | - | *-* | | 1 |  | *I*123 | *t*2 |  | **<=8** | **4** | >8 | >8 | **1** | >2 | **2** | **<=2** | >2/38 |
| 141017135 | *A.junii* | fecal | NDM | - |  | - | - | | 1 |  | *I*123 | *t*12 |  | **<=8** | **<=2** | >8 | >8 | **1** | >2 | **2** | **<=2** | >2/38 |
| 141020016B | *A.junii* | fecal | NDM | - |  | - | *-* | | 1 |  | *I*123 | *t*1 |  | **<=8** | >8 | >8 | >8 | **<=0.5** | >2 | 4 | 8 | >2/38 |
| 141020039 | *A.junii* | fecal | NDM | - |  | - | - | | 1 |  | *I*123 | *t*2 |  | **<=8** | >8 | >8 | >8 | **<=0.5** | **1** | **<=1** | 8 | >2/38 |
| 141020061 | *A.johnsonii* | fecal | NDM | - |  | - |  | *IJK* | - |  | *I*123 | *t*1 |  | **<=8** | >8 | >8 | >8 | **<=0.5** | >2 | 4 | 8 | >2/38 |
| 141020084 | *A.junii* | fecal | NDM | - |  | - | *ABC* |  | 1 |  | *I*123 | *t*2 |  | **<=8** | **<=2** | >8 | >8 | **<=0.5** | >2 | **2** | 8 | >2/38 |
| 141020095 | *A.johnsonii* | fecal | NDM | - |  | - |  | *IJK* | - |  | *I*123 | *t*1 |  | **<=8** | >8 | >8 | >8 | **<=0.5** | >2 | 4 | >8 | >2/38 |
| YQ1027018 | *A.junii* | fecal | NDM | - |  | - | - | | 1 |  | *I*123 | *t*12 |  | **<=8** | **<=2** | >8 | >8 | **<=0.5** | 2 | **2** | **4** | **<=0.5/9.5** |
| YQ1027037 | *A.junii* | fecal | NDM | 58 |  | - | *-* | | 1 |  | *I*123 | *t*12 |  | **<=8** | >8 | >8 | >8 | **<=0.5** | >2 | 4 | 8 | **<=0.5/9.5** |
| YQ1027051 | *A.junii* | fecal | NDM | - |  | - | - | | 1 |  | *I*123 | *t*12 |  | **<=8** | **<=2** | >8 | >8 | **<=0.5** | >2 | **2** | **4** | **2/38** |
| YQ1029087 | *A.**calcoaceticus* | fecal | NDM | - |  | *i* | *ABC* |  | 1 | 5 | *I*123 | *t*2 |  | **<=8** | >8 | >8 | >8 | **<=0.5** | **1** | **<=1** | 8 | >2/38 |
| YQ1029089 | *A.bereziniae* | fecal | - | 24 |  | - | *-* | | 1 |  | *I*123 | *t*2 |  | **<=8** | **<=2** | >8 | >8 | **<=0.5** | **<=0.5** | **<=1** | **<=2** | **<=0.5/9.5** |
| YQ1029097 | *A.junii* | fecal | NDM | 58 |  | - |  | *IJK* | 1 |  | *I*13 | *t*1 |  | **<=8** | 8 | >8 | 8 | **1** | 2 | **<=1** | **<=2** | **<=0.5/9.5** |
| YQ1031098 | *A.junii* | fecal | NDM | - | + | - | - | | 1 |  | *I*123 | *t*1 |  | **<=8** | 8 | >8 | 8 | >2 | >2 | >8 | >8 | >2/38 |
| YQ1031105 | *A.junii* | fecal | NDM | 58 |  | - | - | | - |  | *I*123 |  |  | **<=8** | >8 | >8 | >8 | **<=0.5** | >2 | **2** | 8 | >2/38 |
| YQ1107020 | *A.junii* | fecal | NDM | 23,58 |  | - | - | | 1 |  | *I*123 | *t*12 |  | **<=8** | 8 | >8 | >8 | **<=0.5** | >2 | >8 | 8 | >2/38 |
| YQ1114122B | *A.junii* | fecal | NDM | - |  | - | *-* | | - |  | *I*123 | *t*12 |  | **<=8** | >8 | >8 | >8 | **<=0.5** | >2 | >8 | >8 | >2/38 |
| YQ1114143 | *A.johnsonii* | fecal | NDM | - |  | - |  | *IJK* | 1 |  | *I*123 | *t*12 |  | **<=8** | >8 | >8 | >8 | **<=0.5** | >2 | **<=1** | **<=2** | **<=0.5/9.5** |
| C1119006 | *A.baumannii* | fecal | NDM | 23,51 |  | *b* | *ABC* |  | 1 |  | *I*12 | *t*2 |  | >32 | >8 | >8 | >8 | **<=0.5** | >2 | 8 | >8 | **<=0.5/9.5** |
| C1119024 | *A.baumannii* | fecal | - | 23,51 |  | *b* | *ABC* | *IJK* | - | 5 | *I*123 | *t*1 |  | >32 | >8 | >8 | >8 | **<=0.5** | >2 | >8 | >8 | >2/38 |
| C1119053 | *A.junii* | fecal | NDM | 58 |  | - | - | | 4 |  | *I*123 | *t*2 |  | 32 | >8 | >8 | >8 | **<=0.5** | >2 | **2** | >8 | >2/38 |
| C1119109 | *A.junii* | fecal | NDM | - |  |  | *-* | | - |  | *I*123 | *t*1 |  | 32 | >8 | >8 | >8 | **<=0.5** | >2 | **2** | **<=2** | **<=0.5/9.5** |
| C1121030 | *A.junii* | fecal | NDM  VIM | 58 | + | - | - | | 1 |  | *I*123 | *t*12 |  | **16** | >8 | >8 | >8 | >2 | >2 | 8 | >8 | **1/19** |
| C1121090 | *A.junii* | fecal | NDM | 58 | + | - | *-* | | 1 |  | *I*123 | *t*12 |  | **16** | 8 | >8 | >8 | >2 | >2 | 4 | >8 | >2/38 |
| C1121152 | *A.junii* | fecal | NDM  IMP | 58 | + | - | - | | 1 |  | *I*123 | *t*2 |  | >32 | >8 | >8 | 8 | >2 | >2 | >8 | >8 | **1/19** |
| C1128032 | *A.bereziniae* | fecal | NDM | - | - | - | - | | 2 |  | *I*123 | *t*1 |  | >32 | >8 | >8 | >8 | >2 | >2 | 8 | >8 | >2/38 |
| C1128048 | *A.baumannii* | fecal | - | 24,51 |  | *e* | *ABC* | *IJK* | 1 |  | *I*12 | *t*2 |  | **<=8** | **<=2** | >8 | >8 | **<=0.5** | **<=0.5** | **<=1** | **<=2** | **<=0.5/9.5** |
| C1205001 | *A.baumannii* | fecal | - | 51 |  | *b* | *ABC* | *IJK* | 3 |  | *I*123 | *t*1 |  | >32 | >8 | >8 | >8 | **1** | >2 | >8 | >8 | >2/38 |
| C1205006 | *A.junii* | fecal | NDM  VIM | 58 |  | - | *-* | | 1 |  | *I*123 |  |  | **<=8** | 8 | >8 | 8 | **<=0.5** | >2 | **2** | **<=2** | **<=0.5/9.5** |
| C1208050 | *A.baumannii* | fecal | NDM | 23,51 |  | *b* |  | *IJK* | 1 | 5 | *I*12 | *t*2 |  | >32 | >8 | >8 | >8 | **<=0.5** | >2 | 8 | >8 | >2/38 |
| C1209121 | *A.johnsonii* | fecal | VIM | 58 |  | - |  | *IJK* | 1 |  | *I*123 | *t*1 |  | **<=8** | >8 | >8 | >8 | **<=0.5** | >2 | >8 | >8 | >2/38 |

^1.^ the sequence is shown in Table S3.

^2.^The number of intI inMobile genetic elements : 1, aadA1-catB8-aacA4 ; 2, aacC1-OrfA-OrfB-aadA1; 3, dfrA17-aadA5; 4, aadA2-orfF-dfrA12; 5, dfrA1-sat2-aadA1-orfX( Table S3).

^3..^It including insertion sequences (*I*) and transposons (*t*) . *I*123 mean IS*26*, IS*Aba1* and IS*Aba125. t*12 mean *tnpU* and *tnp513*, respectively.

^4.^AMK, amikacin; GEN, gentamicin; IPM, Imipenem; MEM, Meropenem; CT, colistin; CIP, ciprofloxacin; LVX, levofloxacin; TET, tetracycline; SXT, trimethoprim/sulfamethoxazole. Drug susceptibility according to Clinical and Laboratory Standards Institute (CLSI) M100-S27 criteria; results of susceptible are highlighted in bold.

^5.^a*: a band of approximately 2,000 bp inserts the distal part of the carO porin nucleotide sequences (from 1066 to 2259 of the sequence)
