# Supplementary material for: Molecular characteristics of carbapenem-resistant Acinetobacter spp. from clinical infection samples and fecal survey samples in Southern China
Source: BMC Infect Dis. 2019 Oct 28;19:900. doi: 10.1186/s12879-019-4423-3 (PMC6819553; doi:10.1186/s12879-019-4423-3)
Supplement: Supplementary file 2 — Figure S1. CarO from Acinetobacter species. (a) Homology analysis of CarO amino acids sequences with ATCC 17978. (b) Alignment of CarO amino acids sequences , the CarO amino acids sequences of A6 strain and A592 strain are from NCBI databanks. (DOCX 15 kb) [file 12879_2019_4423_MOESM2_ESM.docx]

**Figure S1**

*CarO* from *Acinetobacter* species. (a) Homology analysis of *CarO* amino acids sequences with ATCC 17978. (b) Alignment of *CarO* amino acids sequences , the *CarO* amino acids sequences of A6 strain and A592 strain are from NCBI databanks.

(a)

(b)
