# Supplementary material for: Molecular characteristics of carbapenem-resistant Acinetobacter spp. from clinical infection samples and fecal survey samples in Southern China
Source: BMC Infect Dis. 2019 Oct 28;19:900. doi: 10.1186/s12879-019-4423-3 (PMC6819553; doi:10.1186/s12879-019-4423-3)
Supplement: Supplementary file 3 — Figure S2. carO gene from Acinetobacter species. (a) Homology analysis of ten novel carO gene sequences with carO gene from ATCC 17978. (b) Alignment of carO gene sequences , the carO gene sequences of A6 strain and A592 strain are from NCBI databanks. (DOCX 124 kb) [file 12879_2019_4423_MOESM3_ESM.docx]

**Figure S2**

*carO* gene from *Acinetobacter* species. (a) Homology analysis of ten novel *carO* gene sequences with *carO* gene from ATCC 17978. (b) Alignment of *carO* gene sequences , the *carO* gene sequences of A6 strain and A592 strain are from NCBI databanks.

(a)

(b)
